# Mapping the DNA Damaging Effects of Polypyridyl Copper Complexes with DNA Electrochemical Biosensors

**DOI:** 10.3390/molecules27030645

**Published:** 2022-01-19

**Authors:** Anna Banasiak, Nicolò Zuin Fantoni, Andrew Kellett, John Colleran

**Affiliations:** 1 Applied Electrochemistry Group, FOCAS Institute, Technological University Dublin, Camden Row, Dublin 8, D08 CKP1 Dublin, Ireland; annabanasiak89@gmail.com; 2 Department of Chemistry, University of Oxford, Oxford OX1 3TA, UK; nicolo.fantonizuin@chem.ox.ac.uk; 3 School of Chemical Sciences and National Institute for Cellular Biotechnology, Dublin City University, Glasnevin, Dublin 9, D09 NR58 Dublin, Ireland; 4 Synthesis and Solid-State Pharmaceutical Centre, School of Chemical Sciences, Dublin City University, Glasnevin, Dublin 9, D09 NR58 Dublin, Ireland; 5 Central Quad Grangegorman, School of Chemical and Pharmaceutical Sciences, Technological University Dublin, Dublin 7, D07 H6K8 Dublin, Ireland

**Keywords:** DNA biosensor, chemical nucleases, DNA-drug interaction, copper complexes, metallodrugs

## Abstract

Several classes of copper complexes are known to induce oxidative DNA damage that mediates cell death. These compounds are potentially useful anticancer agents and detailed investigation can reveal the mode of DNA interaction, binding strength, and type of oxidative lesion formed. We recently reported the development of a DNA electrochemical biosensor employed to quantify the DNA cleavage activity of the well-studied [Cu(phen)_2_]^2+^ chemical nuclease. However, to validate the broader compatibility of this sensor for use with more diverse—and biologically compatible—copper complexes, and to probe its use from a drug discovery perspective, analysis involving new compound libraries is required. Here, we report on the DNA binding and quantitative cleavage activity of the [Cu(TPMA)(*N*,*N*)]^2+^ class (where TPMA = tris-2-pyridylmethylamine) using a DNA electrochemical biosensor. TPMA is a tripodal copper caging ligand, while *N*,*N* represents a bidentate planar phenanthrene ligand capable of enhancing DNA interactions through intercalation. All complexes exhibited electroactivity and interact with DNA through partial (or semi-) intercalation but predominantly through electrostatic attraction. Although TPMA provides excellent solution stability, the bulky ligand enforces a non-planar geometry on the complex, which sterically impedes full interaction. [Cu(TPMA)(phen)]^2+^ and [Cu(TPMA)(DPQ)]^2+^ cleaved 39% and 48% of the DNA strands from the biosensor surface, respectively, while complexes [Cu(TPMA)(bipy)]^2+^ and [Cu(TPMA)(PD)]^2+^ exhibit comparatively moderate nuclease efficacy (ca. 26%). Comparing the nuclease activities of [Cu(TPMA)(phen)] ^2+^ and [Cu(phen)_2_]^2+^ (ca. 23%) confirms the presence of TPMA significantly enhances chemical nuclease activity. Therefore, the use of this DNA electrochemical biosensor is compatible with copper(II) polypyridyl complexes and reveals TPMA complexes as a promising class of DNA damaging agent with tuneable activity due to coordinated ancillary phenanthrene ligands.

## 1. Introduction

Life expectancy has risen significantly during the last few decades due to better accessibility to health care, education, clean water, and sufficient food. During an average lifetime people therefore experience greater exposure to mutagenic substances and suffer age-related losses to cellular function, increasing the chances of permanent DNA damage [1]. It is not surprising then that the number of people diagnosed with cancer is incrementing year-on-year [2]. This trend has emphasised the need for developing effective new anticancer drugs with high specificity, tailored to the individual [3].

Many bioinorganic compounds containing transition metals such as Pt, Ru, Ag, Cu, and Mn exhibit anticancer properties [4,5,6,7,8] with a number undergoing clinical trial [9,10,11]. Among these, copper complexes have proved interesting as their biological activity is closely linked to redox cycling through several oxidation states—Cu(I) and Cu(II) [4]. Their biological and physicochemical properties are also highly dependent on the chelating ligands used in their construction [12,13]. The type and shape of the Cu-coordinated organic scaffold defines the DNA-interaction of the metal complex, which can bind through intercalation, groove binding, or electrostatic interactions [12]. Upon DNA-binding, the metal complex mediates DNA cleavage by hydrolysis of the DNA backbone or by oxidation of DNA sugars or bases [14]. Many copper complexes, in the presence of reductant (ascorbate, glutathione, or NADH) and an oxidant (H_2_O_2_ or O_2_), generate reactive oxygen species (ROS) through Fenton-like [15,16] or other radical-generating mechanisms [17]. These ROS induce damage to DNA, such as the oxidation of bases (e.g., 8-oxoguanine) or to the sugar unit through C–H activation, that result in single- or double-strand DNA breaks [18]. Since the type of DNA damage induced here differs from classical platinum(II) therapy, copper complexes might therefore circumvent existing clinical treatment limitations—especially for recalcitrant cancers of the breast, brain, and pancreas [19,20]. 

The first reported copper-based complex to exhibit oxidative chemical nuclease activity was [Cu(phen)_2_]^2+^ [21]. In recent years, many related copper complexes have been prepared and their chemical nuclease activity explored both biologically and chemically not only in the context of anticancer drug discovery [5,14,22,23], but also for applications in gene-directed cleavage [24,25,26,27] and protein engineering [28]. Chemical nuclease activity is usually examined using gel electrophoresis techniques [29,30,31], further advanced through lab-on-a-chip technology [32]. These methodologies yield valuable insight into the ligand-directed DNA nuclease mechanisms of copper complexes [33]. Attempts to create electrochemical methods to detect DNA cleavage events induced by bioinorganic complexes, have also been reported [34,35,36]. However, these techniques do not provide quantitative data regarding nuclease efficacy and site-specific cleavage. In the current work, the chemical nuclease activities of a new class of copper complex was examined using a quantitative electrochemical method described recently by us [37]. Here, the DNA surface coverage of the biosensor is measured before and after its exposure to the copper nuclease in the presence of reductant and oxidant. Upon DNA cleavage, nucleic acid fragments are released from the immobilised DNA on the electrode surface. This decrease in the DNA surface coverage can be determined electrochemically and presented as the percentage in nuclease efficacy of the complex.

The copper complex class explored herein contain a tris-2-pyridylmethylamine (TPMA) caging ligand and one bidentate ligand selected from: 1,10-phenanthroline (phen); 2,2′-bipyridine (bipy); dipyridoquinoxaline (DPQ); or 1,10-phenanthroline-5,6-dione (PD). TPMA is a tripodal ligand that alone, and as part of a complex with copper, exhibits cytotoxicity against several cancer cell lines [38]. Planar bidentate phenanthrene ligands, on the other hand, are known to interact with nucleic acids by intercalation or semi-intercalation and coordinate a copper ion via *N*,*N* ligation. Importantly, the generation of radical species at the DNA interface by this class leads to strand excision [32,39]. Although the DNA binding and damaging properties of copper, cobalt, and ruthenium complexes of TPMA have been reported [40,41,42,43], these interactions have not, as yet, been characterised using DNA electrochemical biosensors.

In this work, the electrochemical and chemical nuclease properties of copper TPMA complexes were characterised using DNA electrochemical biosensor, recently employed in the analysis of [Cu(phen)_2_]^2+^—an agent which semi-intercalates and cleaves DNA from the minor groove [37]. Herein, the DNA binding and quantitative cleavage activity of the [Cu(TPMA)(*N*,*N*)]^2+^ class is reported using a DNA electrochemical biosensor. These results not only validate the broader compatibility of our DNA sensor for use with polypyridyl complexes, but they also provide valuable drug discovery information related to target identification and selection.

## 2. Results and Discussion

### 2.1. Electrochemical Characterisation of Copper Complexes at Gold Electrodes 

**Scheme 1:**
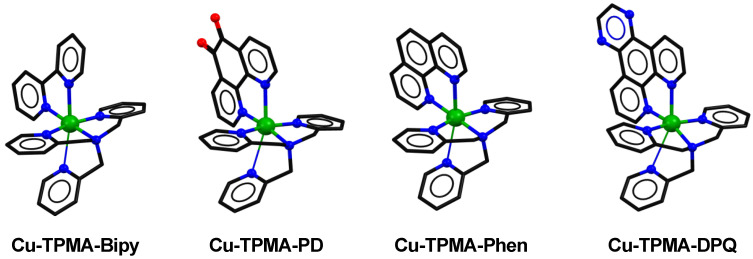
The structures of [Cu(TPMA)(bipy)](ClO_4_)_2_, [Cu(TPMA)(PD)](ClO_4_)_2,_ [Cu(TPMA)(phen)](ClO_4_)_2_ and [Cu(TPMA)(DPQ)](ClO_4_)_2_.

Four copper complexes were examined in this work [44]: [Cu(TPMA)(phen)](ClO_4_)_2_; [Cu(TPMA)(bipy)](ClO_4_)_2_; [Cu(TPMA)(DPQ)](ClO_4_)_2_; and [Cu(TPMA)(PD)](ClO_4_)_2_ (Scheme 1). All complexes were previously characterised using single crystal X-ray analysis and their solution stabilities identified using cw-EPR, HYSCORE, and ENDOR spectroscopies. As shown, the copper centre is bound to four nitrogen atoms of the TPMA ligand and two nitrogen atoms of the bidentate ligand, creating a six-coordinated complex of distorted octahedral geometry [17]. 

**Figure 1:**
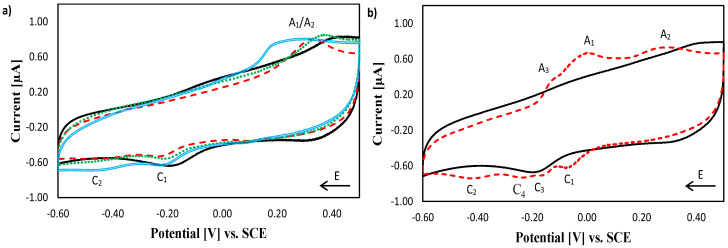
Typical cyclic voltammograms registered in O_2_-free solutions at the bare gold electrode, scan rate 100 mV s^−1^, in 0.1 M PB, pH 7.0 (black solid trace): (**a**) 20 μM of [Cu(TPMA)(phen)]^2+^ (red dashed trace), [Cu(TPMA)(bipy)]^2+^ (blue double trace) and [Cu(TPMA)(DPQ)]^2+^ (green dotted trace), (**b**) 20 μM of [Cu(TPMA)(PD)]^2+^ (red dashed trace).

All copper complexes are electroactive and exhibit an ill-defined electrochemical response at bare gold electrodes in O_2_-free solutions (argon degassed) aqueous solution. The electrochemical profiles obtained for [Cu(TPMA)(phen)]^2+^, [Cu(TPMA)(bipy)]^2+^, and [Cu(TPMA)(DPQ)]^2+^ are very similar (Figure 1a) in that two reduction peaks (C_1_ and C_2_) and one oxidation peak (A_1_) were observed. The electrochemical behaviour of [Cu(TPMA)(PD)]^2+^ is more complicated and several additional oxidation and reduction peaks appear at the bare gold electrode (Figure 1b).

Complications due to the electrochemistry of gold oxide monolayer formation were prevented by removing dissolved oxygen from the solutions. To determine how the individual peaks are correlated with one another, additional measurements in narrower potential windows were performed for [Cu(TPMA)(phen)]^2+^ and [Cu(TPMA)(PD)]^2+^ (S-3, Figures S2–S9, SM). 

In measurements performed for [Cu(TPMA)(phen)]^2+^, it was determined that the C_1_/A_1_ peaks are coupled. The C_2_ redox process occurs only when it is preceded by an A_2_ oxidation event and, thus, the C_2_/A_2_ are also coupled. The C_2_ peak was not observed in our studies of [Cu(phen)_2_]^2+^ [37], hence, its appearance is linked to the presence of the TPMA ligand in [Cu(TPMA)(phen)]^2+^. This was verified by electrochemical characterisation of [Cu(TPMA)]^2+^ under the same conditions which returned redox potential comparable to those reported in polyelectrolyte [45] (S-3, Figure S4, SM). A plausible justification is that after reduction from [Cu(TPMA)(phen)]^2+^ to [Cu(TPMA)(phen)]^+^, C_1_, the complex undergoes structural reorganisation, and subsequent electron transfer, described by the reduction event, C_2_. Elucidation of the mechanism involved here requires further study but may be indicative of dissociation of one of the TPMA chelating N atoms to generate a five-coordinated species [17] (structurally—and possibly chemically—distinct from the initial complex). A similar interpretation can be applied to the C_1_/A_1_ and C_2_/A_2_ redox processes of [Cu(TPMA)(bipy)]^2+^ and [Cu(TPMA)(DPQ)]^2+^. The proposed electrochemical behaviour in aqueous solution justifies the two observed redox couples, referred to hereafter as ‘Cu(TPMA)’ and ‘Cu(*N*,*N′*)’ character, and DNA nuclease properties observed at the DNA modified electrodes, for these complexes.

In measurements performed on [Cu(TPMA)(PD)]^2+^, it was determined that the C_1_/A_1_ redox coupled is ascribed to the ‘Cu(PD) character’ of the complex and C_3_/A_3_ can be associated with the reversible oxidation of the 1,10-phen-5,6-dione (PD) ligand. Transition metal PD complexes can undergo a two-step 2 electron reduction [46,47,48], while the reduction of the free PD ligand in aqueous solution, from quinone to semiquinone and further to hydroquinone, has also been reported [49,50] (S-3, Figure S7, SM). Moreover, the C_2_ peak, also common to the other complexes at −0.4 V, is linked to the A_2_ oxidation processes and describes the electroactivity of the reorganised complex (‘Cu(TPMA) character’).

In general, at both bare and DNA modified gold electrodes, one redox event (C_1_/A_1_), Cu(*N*,*N′*) character’, was recorded at similar potentials for all copper complexes and is comparable to the C_1_/A_1_ data obtained for [Cu(phen)_2_]^2+^ (Table 1), while the C_2_/A_2_ couples describe the ‘Cu(TPMA) character’ redox behaviour of the complexes:

$${\left[\mathrm{Cu}\left(\mathrm{TPMA}\right)\left(N,N\right)\right]}^{2+\;\;}⇌\;\;{\left[\mathrm{Cu}\left(\mathrm{TPMA}\right)\left(N,N\right)\right]}^{+\;}+e^{-}$$

With the exception of [Cu(TPMA)(PD)]^2+^, where reversible redox behaviour is observed in the C_1_/A_1_ couple (∆E_p, **C1/A1**_ = 30 mV), large peak separation values indicate that both C_1_/A_1_ and C_2_/A_2_ redox couples were electrochemically irreversible (∆Ep, **_C1/A1_** > 440 mV and ∆Ep, **_C2/A2_** > 590 mV) at the bare gold electrode. The C_2_ reduction peaks for all copper complexes were registered at similar potentials, whereas the oxidation peaks were observed at positive potential values (>0.23 V). When compared to that of [Cu(phen)_2_]^2+^, it is apparent that the C_1_/A_1_ couple potentials are dictated by the presence of the TPMA ligand. The sluggish A_2_ oxidation processes may indicate that, in terms of complex stability, the reduced state, Cu(I), is favoured, i.e., once reduced, re-oxidation of the complexes is kinetically difficult. These potential values show that differences in the structures of the complexes have a significant bearing on the respective redox properties. 

### 2.2. Electrochemical Characterisation of [Cu(phen)_2_]^2+^, [Cu(TPMA)]^2+^ and the TPMA Ligand at the DNA Biosensor

**Figure 2:**
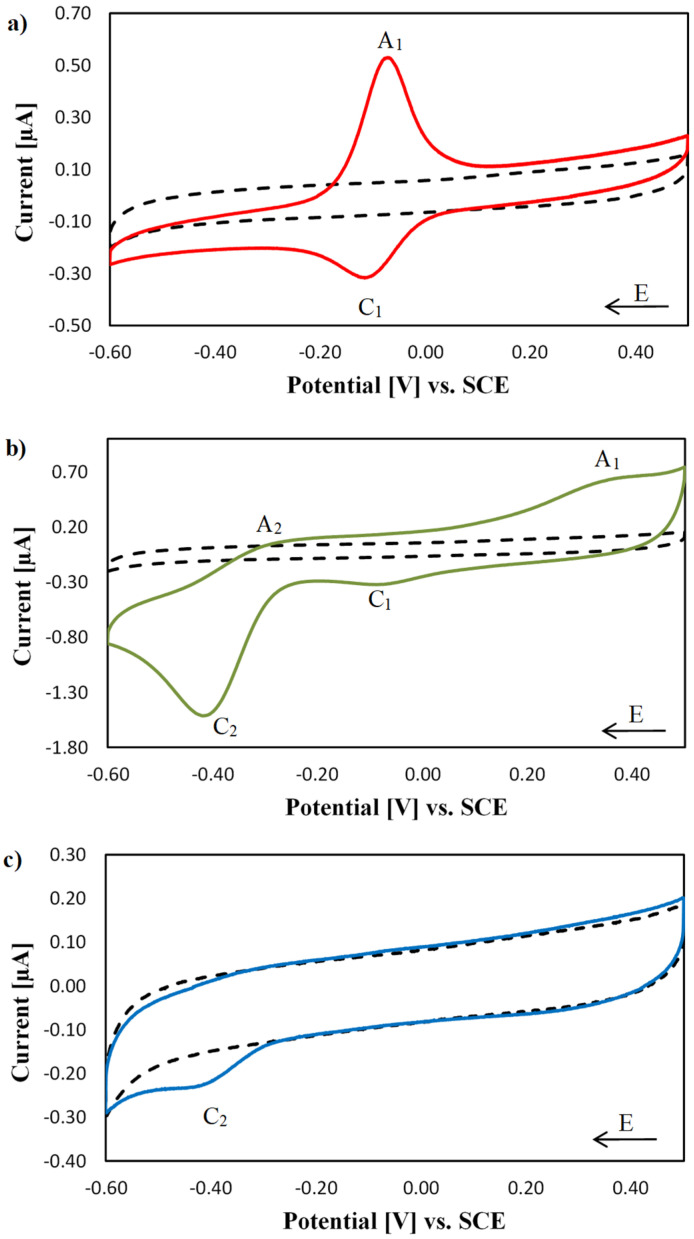
Typical cyclic voltammograms, scan rate 100 mV s^−1^, registered at the DNA biosensor in 0.1 M PB, pH 7.0 (black dashed trace), and in 20 µM solutions of (**a**) [Cu(phen)_2_]^2+^ (red solid trace), (**b**) [Cu(TPMA)]^2+^ (green solid trace) and (**c**) TPMA ligand (blue solid trace). Scan rates, 100 mV s^−1^.

To interpret the data obtained for [Cu(TPMA)(*N*,*N′*)]^2+^, electrochemical analysis of [Cu(phen)_2_]^2+^, [Cu(TPMA)]^2+^ and the TPMA ligand at the DNA biosensor was carried out (Figure 2). The redox peaks for [Cu(phen)_2_]^2+^ were clearly visible at −0.12 V (C_1_) and −0.07 V (A_1_) vs. SCE (Figure 2a). This redox process can be associated with the reduction and oxidation of the complex, [Cu(phen)_2_]^2+^/[Cu(phen)_2_]^+^, mediated by the immobilised DNA layer via long-range electron transfer. A peak potential separation, ∆Ep, of 50 mV, and the ratio between the oxidation and reduction peak currents, I_p,a_/I_p,c_ > 2, indicate that the redox reaction is quasi-reversible. The electrochemistry of this compound is discussed in detail elsewhere. An ill-defined C_1_/A_1_ redox couple was evident for [Cu (TPMA)]^2+^ (Figure 2b). The small reduction C_1_ peak appears at a potential similar to that for [Cu(phen)_2_]^2+^ but the oxidation A_1_ peak is shifted to a much more positive potential value. The differences in the redox potentials between the complexes are directed by the variations in complex structure and ligand type. The planar aromatic ligand of [Cu(phen)_2_]^2+^ facilitates the partial intercalation of the complex within DNA bases [51] and the complex redox reaction is then mediated via long-range electron transfer.

The bulky non-planar TPMA ligand impedes intercalation between DNA bases, precluding close contact between the complex and DNA; hence, the electrochemical response of this complex is less well-defined than that observed for [Cu(phen)_2_]^2+^. An additional reduction peak C_2_, at −0.42 V vs. SCE, (not observed in [Cu(phen)_2_]^2+^) can be linked to the presence of TPMA ligand in the complex, confirmed by the single small reduction peak (C_2_) at −0.40 V vs. SCE for the TPMA ligand alone (Figure 2c). The presence of this peak indicates that the TPMA ligand can be reduced via DNA-mediated electron transfer. 

The TPMA ligand consists of three pyridine rings that can, in theory, be reduced when free in solution [52]. The pyridine ring (pKa 5.2 [53]) after protonation in PB, pH 7.0, can undergo a one electron reduction to the pyridinyl radical (S-4, Figure S10, SM). Finally, the C_2_ peak current was ca. 15 times larger for [Cu(TPMA)]^2+^ than for the uncharged TPMA ligand alone, for the same concentration of compounds—evidence of a synergistic effect between the redox active copper and TPMA ligand, coupled to electrostatically-facilitated complex interaction with the immobilised DNA strands.

### 2.3. Electrochemical Characterisation of [Cu(TPMA)(N,N′)]^2+^ Complexes at the DNA Biosensor

**Figure 3:**
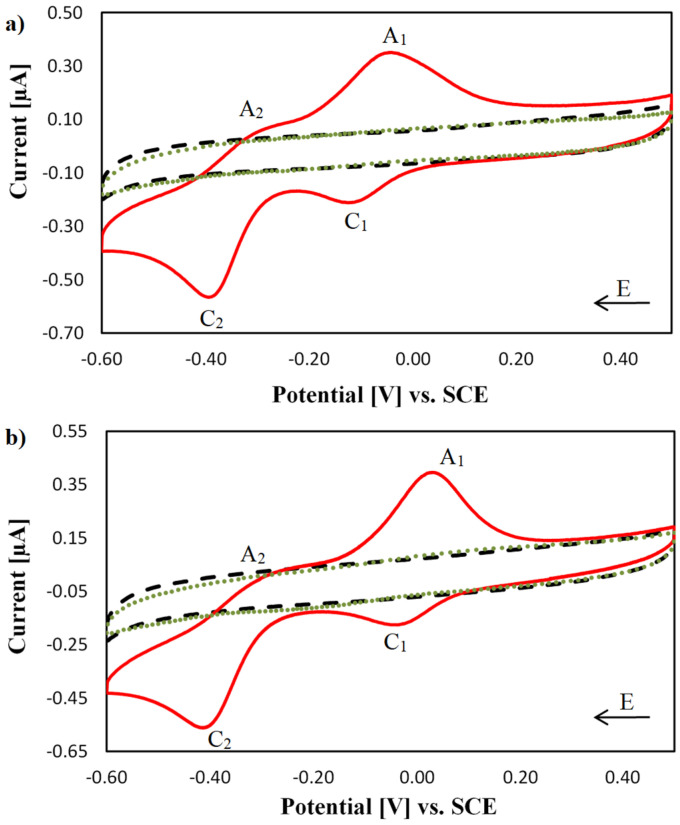
Typical cyclic voltammograms registered at the DNA biosensors, in 0.1 M PB, pH 7.0, scan rate 100 mV s^−1^ (black dashed trace), in 20 µM of (**a**) [Cu(TPMA)(phen)]^2+^ (**b**) [Cu(TPMA)(DPQ)]^2+^ (red solid trace) and after washing the complex from the DNA layer for 1 h (green dotted trace).

Two prominent redox couples (C_1_/A_1_ and C_2_/A_2_) were observed for [Cu(TPMA)(phen)]^2+^ and [Cu(TPMA)(DPQ)]^2+^ at the DNA biosensor (Figure 3). Analysis of the peak potentials (S-5, Table S1, SM) indicate that the C_1_/A_1_ redox processes are quasi- reversible, while the C_2_/A_2_ processes tend toward irreversibility [54]. The C_1_/A_1_ redox waves, observed for [Cu(TPMA)(phen)]^2+^ and [Cu(phen)_2_]^2+^, occur at similar potentials indicating that both are related to the same redox event, i.e., representing the ‘[Cu(phen)_2_]^2+^ electronic character’ of the [Cu(TPMA)(phen)]^2+^ complex. The C_2_/A_2_ wave was observed for all copper complexes except for [Cu(phen)_2_]^2+^, which suggests that the redox wave observed at these potentials is directed by the presence of the TPMA ligand. In support of this assignment, the C_2_ reduction peak appears at very similar potentials for [Cu(TPMA)]^2+^ and [Cu(TPMA)(*N*,*N′*)]^2+^ at the DNA biosensors. Thus, the C_2_/A_2_ redox wave is associated with the ‘[Cu(TPMA)]^2+^ electronic character’ of the complex. Copper complexes containing TPMA have shown solvent-dependent coordination spheres—five-coordinate in H_2_O (no salts present) and mixed five and six-coordinate in organic solvent [17]. Data generated at the DNA modified electrodes seem to indicate that the complexes are six-coordinate in phosphate buffer and the dissociation of one Cu–N dative bond in aqueous solution generates a single unoccupied catalytic site within the complexes. This structural reorganisation would seem to justify the distinct mixed redox behaviour observed in these complexes at DNA modified electrodes. 

The interactions between DNA and the complexes were next evaluated through the changes in the E^0′^ values for the C_1_/A_1_ and C_2_/A_2_ redox couples at the bare and DNA modified electrodes (S-5, Table S2, SM). Similar analysis using the C_2_/A_2_ redox couple could not be conducted since the A_2_ peak was not evident at bare gold electrodes and a formal potential could not be estimated. The negative shift in the C_1_/A_1_ formal potential, observed in both [Cu(TPMA)(phen)]^2+^ and [Cu(TPMA)(DPQ)]^2+^, indicate that these complexes interact with DNA predominantly through electrostatic interactions in these conditions [55]. However, the presence of the C_1_/A_1_ redox couple observed at similar potentials for [Cu(TPMA)(phen)]^2+^ and [Cu(phen)_2_]^2+^ indicates that, despite steric hindrance impeding intercalation, [Cu(TPMA)(phen)]^2+^ is positioned in close enough proximity to the DNA strands to undergo DNA mediated redox reactions.

The [Cu(TPMA)(DPQ)]^2+^ C_1_/A_1_ redox couple is shifted to less negative potentials compared to those observed with [Cu(TPMA)(phen)]^2+^, and [Cu(TPMA)(PD)]^2+^, inferring a more facile redox reaction than for [Cu(TPMA)(phen)]^2+^. The planar aromatic ligand, DPQ, is of higher aromatic surface area than phenanthroline, phendione, and bipyridine; hence, despite the bulky TPMA ligand, the complex can intercalate between DNA bases to a greater extent than the phenanthroline, phendione, and bipyridine analogues, placing the copper centre in closer proximity to the DNA strands. This effect corroborates earlier topoisomerase unwinding experiments where the DPQ complex unwound superhelical plasmid DNA in manner similar to the classical DNA intercalator ethidium bromide while the phenanthroline complex was not effective [17].

**Figure 4:**
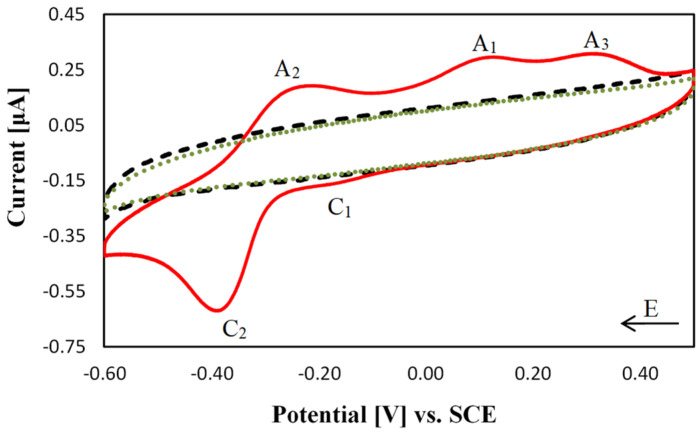
Typical cyclic voltammograms registered at the DNA biosensor in 0.1 M PB, pH 7.0 (black dashed trace), in 20 µM of [Cu(TPMA)(bipy)]^2+^ (red solid trace) and after washing [Cu(TPMA)(bipy)]^2+^ from the DNA layer for 1 h (green dotted trace). Scan rates, 100 mV s^−1^.

The disappearance of [Cu(TPMA)(phen)]^2+^ and [Cu(TPMA)(DPQ)]^2+^ redox waves, after washing the DNA layer for one hour in 0.1 M PB (0.5% ACN), pH 7.0, confirms that the interactions between the complexes and DNA are non-covalent in character (Figure 3; green dotted traces). The 2,2′-bipyridine ligand (bipy) is known to interact with DNA through minor groove binding [56,57]; however, if more than one bipy ligand is present in the complex, the complex tends to interact with DNA through electrostatic attraction, presumably due to steric hindrance caused by the size of the complex [55,58]. The electrochemical profile for [Cu(TPMA)(bipy)]^2+^ was expected to be then of a slightly different character than complexes presented above. The electrochemical response registered at the DNA biosensor for [Cu(TPMA)(bipy)]^2+^ reveals the presence of C_1_/A_1_ and C_2_/A_2_ redox couples and an additional oxidation peak A_3_ (Figure 4). The C_1_ peak is ill-defined compared to that observed for [Cu(TPMA)(phen)]^2+^ and [Cu(TPMA)(DPQ)]^2+^ indicating a less facile reduction reaction occurs in [Cu(TPMA)(bipy)]^2+^. Moreover, the C_1_/A_1_ redox process appeared to be irreversible in contrast to the results obtained for [Cu(TPMA)(phen)]^2+^ and [Cu(TPMA)(DPQ)]^2+^ (Table S2).

Therefore, it appears that the 2,2’-bipyridine ligand cannot position the copper metal centre in close proximity to DNA, while 1,10-phenanthroline and DPQ—even if only through partial intercalation—can position the metal centre close enough to facilitate redox reactions. The additional A_3_ oxidation peak appeared at +0.30 V vs. SCE, at a similar potential to the A_1_ peak of [Cu(TPMA)]^2+^ at the DNA biosensor (S-5, Table S1, SM). This result suggests that the A_3_ redox peak is of ‘[Cu(TPMA)]^2+^ character’. Moreover, the A_2_ peak, although still irreversible, was much more prominent in [Cu(TPMA)(bipy)]^2+^ than in [Cu(TPMA)(phen)]^2+^ and [Cu(TPMA)(DPQ)]^2+^, indicating again that the ‘[Cu(TPMA)]^2+^ character’ presents the dominant redox process in [Cu(TPMA)(bipy)]^2+^. The negative shift in the E^0′^ in the presence of DNA compared to the E^0′^ obtained in the absence of DNA (S-5, Table S2, SM) suggests that [Cu(TPMA)(bipy)]^2+^ interacts with DNA predominantly through electrostatic attraction. Additionally, the lack of any faradaic processes at the DNA biosensor after washing indicates that the complex interacts with DNA in a non-covalent manner.

**Figure 5:**
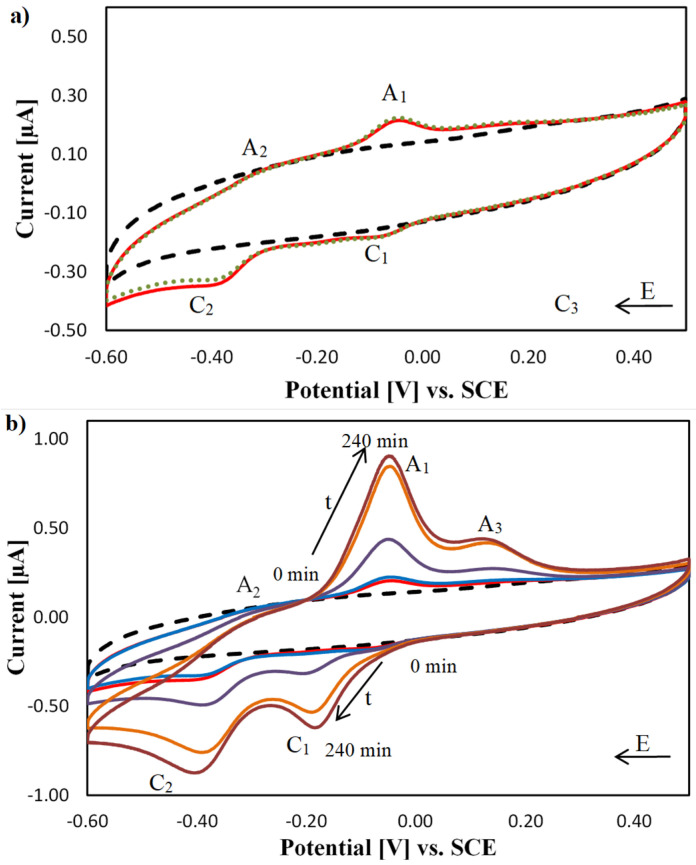
Typical cyclic voltammograms, obtained at 100 mV s^−1^ scan rates, registered at the DNA biosensors in 0.1 M PB, pH 7.0 (black dashed trace), and in 20 µM of [Cu(TPMA)(PD)]^2+^ (**a**) just after addition (red solid trace) and after 60 min (green dotted trace); (**b**) over time using a single DNA sensor: 0 min (red trace); 60 min (blue trace); 120 min (violet trace); 180 min (orange trace); and 240 min (brown trace).

Analysis of the electrochemical profile of [Cu(TPMA)(PD)]^2+^ revealed time-dependent changes. The redox couples C_1_/A_1_ and C_2_/A_2_ observed for [Cu(TPMA)(PD)]^2+^ at the DNA biosensor were initially much smaller in magnitude than redox peaks observed with the other complexes (Figure 5a). The C_1_/A_1_ redox wave was recorded at similar potentials to those at the bare gold electrode (C_1_ at −0.08 V and A_1_ at −0.04 V vs. SCE) and can be associated with the electrochemical reduction of the complex [48], directed by PD electronic characteristics.

The C_2_/A_2_ redox couple, the ‘[Cu(TPMA)]^2+^ character’ of the complex, was evident −0.42/−0.33 V vs. SCE. After 60 min, at the same DNA sensor, the peaks expanded and shifted over time (Figure 5b). Moreover, an additional peak (A_3_) at +0.14 V became evident. The changes in the electrochemical behaviour of the complex could be due to disruption of the DNA layer and further insertion of the complex within the DNA strands. 

The [Cu(TPMA)(PD)]^2+^, in contrast to the other studied complexes, was not easily removed by washing from the DNA layer. Some [Cu(TPMA)(PD)]^2+^ redox activity was recorded at the DNA biosensor in pure PB up to two days after exposure of the biosensor to the [Cu(TPMA)(PD)]^2+^ solution (S-6, Figure S11, SM). Despite washing, the presence of the complex in the layer for an extended period of time suggests that the complex interacts with DNA in a covalent manner. According to the literature, some quinones can covalently bind to DNA bases, creating DNA adducts [59,60].

While fresh DNA sensors were prepared for the characterisation of each complex, scan rate experiments were performed at single DNA sensors and presented results are representative of typical analyses. These data were repeatable between freshly made DNA sensors. The relationship between the oxidation and reduction peak currents and the square root of scan rate, for each copper complex at the DNA biosensor, was linear while the relationship between peak current and scan rate was not (S-7, Figures S12–S15, SM). The redox reactions of the presented copper complexes were then deemed to be under diffusion control. In contrast, the redox reaction of [Cu(phen)_2_]^2+^, measured under the same conditions at the DNA biosensors, is under adsorption control [37]. This indicates that the presence of the bulky cage ligand, TPMA, directs a diffusion-controlled process and confirms that intercalation within the immobilised DNA strands is effectively prevented. The shift in estimated formal potentials are all negative for the complexes at the DNA biosensors, indicative of electrostatic interaction regimes (S-5, Table S2, SM). The negative shift in the C_2_/A_2_ formal potentials follow the trend Cu(TPMA)(DPQ)]^2+^ < [Cu(TPMA)(phen)]^2+^ < [Cu(TPMA)(PD)]^2+^ < [Cu(TPMA)(bipy)]^2+^ which would seem to reflect expected DNA intercalative properties of the ancillary *N*,*N′* ligands.

The electrochemical profiles for the copper complexes were also registered using square wave voltammetry. These data were used to check the stability of copper complex interaction with DNA. Prolonged exposure of the complexes [Cu(TPMA)(phen)]^2+^, [Cu(TPMA)(DPQ)]^2+^ and [Cu(TPMA)(bipy)]^2+^, to the DNA biosensors revealed that the redox peaks were stable indefinitely, while a marked changed was observed for [Cu(TPMA)(PD)]^2+^ after one hour (S-8, Figures S16–S19, SM).

### 2.4. DNA Nuclease Efficacy of Copper Complexes

Double-strand DNA breaks (DSBs) mediated by the copper complexes in the presence of an exogenous oxidant and reductant were monitored electrochemically using the DNA biosensors. The biosensors do not distinguish independent DSBs from proximate single strand breaks (SSBs) leading to double strand cleavage and in this assay, detection of the latter is likely given our earlier nicking observations by [Cu(TPMA)(*N*,*N*)]^2+^ complexes with supercoiled pUC19 DNA [17]. The DNA surface coverages of the DNA biosensors were measured, prior to exposure to the copper complex nuclease assays, in 10 mM Tris-HCl, pH 7.4, using RuHex as a redox probe [37,61]. The DNA biosensors were then exposed to a nuclease assay containing 10, 20, and 50 µM of the complex, 1 mM ascorbic acid (AA) as the reductant and 1 mM H_2_O_2_ as the oxidant in 0.1 M PB, pH 7.0, at 37 °C for two hours. The DNA surface coverage was measured again after cooling and washing the DNA biosensor. The DNA layer washing is essential to ensure that the components of the nuclease assay are removed from the DNA layer and do not affect the subsequent interactions between DNA and RuHex. The changes in the DNA surface coverage values are then the consequence of DNA cleavage from the electrode surface. The cleavage efficacy (*C*.*E*.) can be then calculated as shown in Equation (1):

(1) $$C.E.=100\;\%-\left(\frac{\Gamma_{DNA\;after\;nuclease\;assay}}{\Gamma_{DNA\;before\;nuclease\;assay}}\times 100\%\right)$$

Since the Oligo DNA was 30 base pairs long, the number of cleaved base pairs was calculated as follows, Equation (2):

(2) $$BP\;Cleaved=30\;\mathrm{bp}-\left(\frac{\Gamma_{DNA\;after\;nuclease\;assay}}{\Gamma_{DNA\;before\;nuclease\;assay}}\times 30\;\mathrm{bp}\right)$$

**Figure 6:**
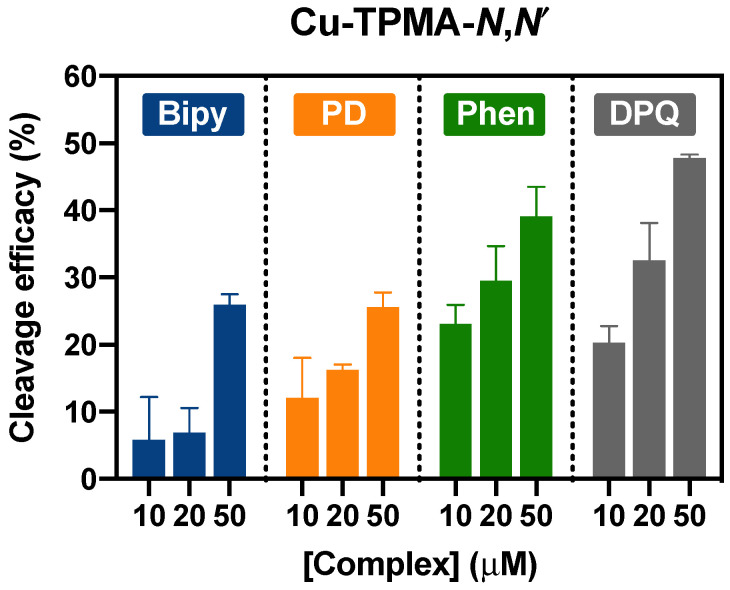
Average DNA nuclease efficacies determined at DNA modified electrodes for the copper complexes in 10, 20, and 50 µM concentrations; [Cu(TPMA)(bipy)]^2+^ (blue bars), [Cu(TPMA)(PD)]^2+^ (orange bars), [Cu(TPMA)(phen)]^2+^ (green bars), and [Cu(TPMA)(DPQ)]^2+^ (grey bars).

The nuclease activities of all copper complexes at concentrations of 10, 20, and 50 µM are presented in Figure 6. Individual data sets for the nuclease and control experiments, with %RSD values, are available in the supporting information (S-9, Tables S3–S6, SM). All measurements were performed in triplicate using DNA biosensors freshly prepared for each measurement. The control measurements for this method, namely, the oxidant alone, the reductant alone and a mixture of the reductant and oxidant have no effect on the DNA surface coverage and are published elsewhere [37]. It was determined that the presence of a small amount of ACN (up to 1% *v*/*v* checked, S-2, Figure S1, SM) in the solution did not affect the DNA surface coverage values. Moreover, the complexes alone, in the absence of an exogenous reductant and oxidant, did not cause DNA cleavage.

[Cu(TPMA)]^2+^ was found to cause insignificant DNA cleavage, 5.36% (%RSD 7.50%)—estimated as one base pair, in the presence of an exogenous reductant and oxidant. The non-planar geometry of TPMA dictates the proximity of the copper centre of the complex to the DNA strands. A relatively large distance between the copper centre and the DNA strands limits the ability to cleave DNA as the potency and frequency of radical species generated by the complex are greatly diminished. This result is in good agreement with results already published for this complex. Humphreys et al. first examined the nuclease activity of [Cu(TPMA)]^2+^ using plasmid DNA and radiolabelled sequences [40,41]. In that work, the complex did not cause double-strand breaks in any of the tested DNA types; however, [Cu(TPMA)]^2+^ did cause single-strand breaks in the plasmid. The same effect was observed by us on plasmid DNA [17] and in the current study it is likely proximate single strand breaks by [Cu(TPMA)]^2+^ gives rise to the observed base pair cleaved. In contrast to this finding, the [Cu(TPMA)(*N*,*N*)]^2+^ complexes exhibited high cleavage activity under the same conditions. Planar aromatic ligands in complexes enhance the binding activity between DNA and complex [17] and the redox properties of the complex (different E^0′^ values for different complexes), having a marked effect on the observed nuclease efficacies. Previous studies have shown that superoxide radical and hydrogen peroxide generation play main roles in the DNA damage induced by the [Cu(TPMA)(*N*,*N*)]^2+^ family. Moreover, some evidence of DNA damage associated with deamination of adenine (via the creation of deoxyinosine) was evident [17].

In this work, [Cu(TPMA)(phen)]^2+^ exhibited high nuclease activity, even in small concentrations (S-9, Table S3, SM). Comparatively, 10 µM [Cu(TPMA)(phen)]^2+^ cleaved as much DNA from the electrodes as 50 µM of [Cu(phen)_2_]^2+^ (23%) under the same experimental conditions [37]. The higher nuclease activity of [Cu(TPMA)(phen)]^2+^ is likely due to the presence of the TPMA ligand enhancing the complex solution stability. The ligand also dictates the oxidation potential of the complex through stabilisation of the Cu(I) oxidation state (Section 3.1), serving to promote the generation of ROS and, ultimately, enhanced nuclease efficacy in comparison to [Cu(phen)_2_]^2+^. This effect was evident for the family of [Cu(TPMA)(*N*,*N′*)]^2+^ analogues presented here.

[Cu(TPMA)(DPQ)]^2+^ in the presence of the reductant and oxidant exhibited the highest nuclease efficacy of all complexes (S-9, Table S4, SM). A concentration of 50 µM [Cu(TPMA)(DPQ)]^2+^ cleaves almost half of the DNA strands present on the electrode surface. The nuclease activity of a copper complex containing a DPQ ligand, [Cu(DPQ)_2_(H_2_O)]^2+^, was also reported by Santra et al. [62], with the cleavage of plasmid DNA observed on an agarose gel. The DNA degradation was more extensive for [Cu(DPQ)_2_(H_2_O)]^2+^ than for the same concentration of [Cu(phen)_2_(H_2_O)]^2+^ (non-quantitative data). Following the logic attributed to [Cu(TPMA)(phen)]^2+^, the extended phenanthrene DPQ ligand should penetrate further into the DNA base pairs placing the complex in closer proximity to the DNA strands, facilitating more efficient DNA mediated charge transfer. Enhanced redox cycling leading to efficient in situ generation of ROS results in a complex with potent DNA cleavage properties. 

The [Cu(TPMA)(bipy)]^2+^ complex did not exhibit significant DNA cleavage at concentrations of 10 μM and 20 μM (S-9, Table S5, SM). Moderate cleavage was observed only when a 50 μM concentration was used. These results suggest that an excess of the complex is necessary to cause double-strand breaks (again resulting from proximate SSBs). According to the literature, copper complexes with 2,2′-bipyridine do not possess significant DNA nuclease properties [15,63]. The presence of the TPMA ligand appears to enhance complex cleavage efficacy to some degree, likely due enhanced solution stability leading to the creation of a favourable redox environment, promoting radical formation.

[Cu(TPMA)(PD)]^2+^ did not exhibit nuclease activity in the absence of exogenous oxidant and reductant (S-9, Table S6, SM). However, the electrochemical profile of [Cu(TPMA)(PD)]^2+^ registered at the DNA biosensors (in the absence of exogenous oxidant and reductant) reveals that the complex disrupts the DNA duplex significantly over time (Figure 5). The observed changes in the oxidation and reduction peaks then are not associated with DNA ablation from the electrode surface but are likely representative of a covalent DNA binding process. Conversely, in the presence of the exogenous reductant and oxidant, [Cu(TPMA)(PD)]^2+^ exhibits moderate and concentration dependent DNA cleavage.

## 3. Materials and Methods

### 3.1. Materials

[Cu(phen)_2_](NO_3_)_2_ and the copper complexes of the general formula: [Cu(TPMA)(*N*,*N′*)]^2+^ were synthesised as reported [17,44]. The [Cu(TPMA)(*N*,*N′*)]^2+^ complexes were dissolved in acetonitrile (ACN) prior to further dilution in phosphate buffer (PB). The final concentration of ACN in the PB solutions of the complexes did not exceed 0.2% (*v*/*v*). Ruthenium(III) hexaamine trichloride 98% and 4-mercaptotoluene 98% were purchased from Fisher. All other chemicals were of analytical grade and were purchased from Sigma–Aldrich. DNA oligomers containing 30 nucleotides were also purchased from Sigma–Aldrich and had the following sequences:

Thiol-modified strand: SH-(CH_2_)_6_- 5’-AGTACAGTCATCGCTTAATTATCGTACGTA-3’ Complementary strand: 3’-TCATGTCAGTAGCGAATTAATAGCATGCAT-5’.

DNA strands were hybridised prior to use according to the procedure provided by Sigma–Aldrich, Arklow, Ireland. Argon gas of technical grade was purchased from Air Products. Nuclease-free water was used for the preparation of the oligo DNA solutions, while Milli-Q^®^ water was used to prepare all other solutions. 

### 3.2. Equipment

The electrochemical measurements were performed with a CH Instruments, Inc. (IJ Cambria Scientific Ltd., Llanelli, UK) potentiostat, model 620A. DNA-modified gold electrodes (2 mm diameter, from CH Instruments, Inc.), a saturated calomel electrode (SCE, from BAS Inc., West Lafayette, IN, USA) and a platinum wire (from Surepure Chemetals, Florham Park, NJ, USA) were used as the working, reference, and counter electrodes, respectively. The preparation of the DNA-modified gold electrodes (DNA biosensors) is described in S-1, Supplementary Materials (SM). All glassware in contact with DNA was silanised to offset adsorption of DNA from solutions [64]. Prior to DNA immobilisation, bare gold electrodes were cleaned by manual polishing with a 0.05 μm Al_2_O_3_ slurry on Buehler microcloth for 5 min, followed by electrochemical cycling in 0.5 M deaerated H_2_SO_4_, over the potential range −0.2 V to +1.5 V vs. SCE at a scan rate of 100 mV s^−1^, until steady-state current was attained. The thiol-modified gold electrodes were cleaned by electrochemical desorption of thiols through cycling in 0.5 M KOH, over the potential range +0.1 V to −1.4 V vs. SCE at a scan rate of 50 mV s^−1^, followed by manual polishing on microcloth with 0.05 μm Al_2_O_3_ for 5 min. The supporting electrolyte was deoxygenated with argon before measurements and a blanket of argon was maintained above the solutions during measurements. The electrochemical measurements were performed at room temperature, while the chemical nuclease assays were carried out at 37 °C.

### 3.3. Interactions between Immobilised DNA and Bioinorganic Compounds

The interactions between the complexes and DNA were deciphered through analysis of the electrochemical profile of the compound in the absence and presence of DNA [55]. If the E^0’^ value, estimated as the mean peak potential for the redox couple of the complex, shifts to more positive potentials in the presence of DNA, the complex was interpreted to interact with DNA predominantly through intercalation. If the E^0’^ value shifts to more negative potentials, the interactions between DNA and the compound was interpreted as predominantly electrostatic.

### 3.4. Determination of DNA Surface Coverage

The DNA surface coverage was measured electrochemically using ruthenium(III) hexaamine (RuHex) as a redox probe [37,61,65]. In low ionic strength electrolyte (10 mM Tris-HCl), the positively charged RuHex interacts with DNA electrostatically, as an external binder, replacing the existing phosphate backbone counterions (Na^+^ or K^+^). The amount of RuHex molecules adsorbed in the DNA strands can be determined electrochemically using chronocoulometry. Upon DNA layer saturation, the number of adsorbed RuHex molecules is proportional to the number of phosphate groups in the DNA strands. Hence, knowing the number of phosphate groups present in the custom DNA strands (one nucleotide contains one phosphate group), the amount of adsorbed RuHex can be related to the number of DNA molecules immobilised at the electrode surface. After each aliquot addition of RuHex, and prior to data acquisition, the solution was allowed to equilibrate with the DNA sensor for two minutes.

### 3.5. Electrochemical Measurements and Nuclease Assay Conditions

Each measurement was performed independently on freshly prepared electrodes in 0.1 M phosphate buffer (PB), pH 7.0, containing 20 µM of the complex. Cyclic voltammetry was carried out over the potential range +0.5 V to −0.6 V vs. SCE, at a scan rate of 0.1 V s^−1^. The [Cu(TPMA)(*N*,*N′*)]^2+^ solutions were allowed to equilibrate with the DNA sensors for 20 min prior to characterisation using cyclic voltammetry. To promote solubility in PB, the complexes were first dissolved in ACN, and aliquots were then added to PB (amount of ACN in PB was 0.2% *v*/*v*). The addition of ACN to PB does not affect the integrity of the DNA layer (S-2, Figure S1, SM). Chronocoulometry was then performed over the potential range +0.1 V to −0.6 V vs. SCE with a pulse width of 0.5 s. The DNA biosensor was exposed to the DNA nuclease assay, comprising of 10 µM, 20 µM, or 50 µM of the chosen complex, 1 mM of sodium-*L*-ascorbate (AA) and 1 mM of H_2_O_2_ for two hours at 37 °C.

## 4. Conclusions

The copper complexes investigated in this work were all found to be electroactive and interact with DNA predominantly through electrostatic attraction. The intercalation of planar aromatic bidentate ligands, such as 1,10-phenanthroline and DPQ, was hindered due to the bulky TPMA scaffold. The electrochemical response of [Cu(TPMA)(bipy)]^2+^ was dominated by the ‘[Cu(TPMA)]^2+^ character’ of the complex. It was also found that [Cu(TPMA)(PD)]^2+^ caused strong disruption of the DNA layer over time and remains within the DNA layer for an extended period of time even after washing. Dissociation was not observed in any of studied complexes under our experimental conditions, which corroborates earlier EPR results. Detailed investigations into the nuclease activity toward immobilised DNA strands provided an interesting array of results. Firstly, in the presence of an exogenous reductant and oxidant, [Cu(TPMA)(phen)]^2+^ and [Cu(TPMA)(DPQ)]^2+^ displayed relatively high nuclease activity by cleaving 39% and 48% of the DNA strands at 50 μM drug-loading, respectively. Under the same conditions, [Cu(TPMA)(bipy)]^2+^ and [Cu(TPMA)(PD)]^2+^ exhibit moderate nuclease efficacy with ca. 26% cleavage activity detected for both agents. Although the [Cu(TPMA)]^2+^ complex alone displays poor chemical nuclease activity, coordination of phen or DPQ promotes excellent cleavage ability. The type of ancillary ligand present also significantly affects both the DNA binding activity and oxidative DNA damage induced. Although electrostatic forces dominate DNA-[Cu(TPMA)(*N*,*N*)]^2+^ interactions, the intercalative properties of phen and DPQ facilitate greater accessibility to DNA bases. Consequently, greater electronic communication to the DNA base pairs grant [Cu(TPMA)(phen)]^2+^ and [Cu(TPMA)(DPQ)]^2+^ enhanced DNA cleavage abilities, through more efficient DNA mediated redox cycling, and concomitant in situ generation of reactive oxygen species. This assertion may seem to contradict data gleaned for [Cu(TPMA)(PD)]^2+^, but based on electrochemical behaviour observed at the DNA biosensors in the absence of exogenous reagents, we postulate that PD-initiated DNA coupling reactions occur that compete with the DNA oxidative damage pathway, limiting the nuclease efficacy observed for this complex. Overall, DNA electrochemical biosensors appear highly suitable for analysing copper polypyridyl complexes and can provide valuable structure-activity-relationship (SAR) data from both DNA damage and drug discovery perspectives. It appears TPMA provides strong solution stability that stabilises the Cu(I) oxidation state of this complex series thereby facilitating the generation of reactive oxygen species. Despite the predominant electrostatic interaction of [Cu(TPMA)(phen)]^2+^ with DNA, its nuclease activity was higher than the known DNA semi-intercalator, [Cu(phen)_2_]^2+^. The results obtained here clearly show how the ligand scaffold and ancillary *N*,*N* ligands potentiate both the redox and chemical nuclease properties of bioinorganic copper complexes.

## Figures and Tables

**Table 1:** The values of C_1_/A_1_ and C_2_/A_2_ redox couple anodic and cathodic peak potentials at bare gold electrodes.

Complex	Ep,_C1_/Ep,_A1_ [V]	ΔEp,_C1/A1_ [mV]	Ep,_C2_/Ep,_A2_ [V]	ΔEp,_C2/A2_ [mV]
[Cu(TPMA)(Phen)]^2+^	−0.18/+0.30	480	−0.46/+0.30	760
[Cu(TPMA)(DPQ)]^2+^	−0.21/+0.23	440	−0.46/+0.23	590
[Cu(TPMA)(Bipy)]^2+^	−0.19/+0.35	540	−0.45/+0.35	800
[Cu(TPMA)(PD)]^2+^	−0.07/−0.04	30	−0.43/+0.27	700
[Cu(TPMA)]^2+^	−0.21/+0.01	220	−0.42/+0.22	640
^✻^ [Cu(Phen)_2_]^2+^	−0.20/−0.08	120	NA	NA

Scan rate 100 mV s^−1^, in 0.1 M PB, and the separation between these potentials, ∆Ep. ^✻^ From reference [37].

## Data Availability

The data presented in this study are available in this article and supplementary material.

## Supplementary Materials

The following are available online. Figure S1: The effect of acetonitrile on the DNA layer of the DNA biosensor; Figures S2–S6, S8, S9: Electrochemical response of [Cu(TPMA)(phen)]_2+_, [Cu(TPMA)]_2+_, and [Cu(TPMA)(PD)]_2+_ at the gold electrode; Figure S7: Scheme of quinone electrochemical reduction; Figure S10: Reduction of the pyridine ring; Tables S1 and S2: Electrochemical parameters for the copper complexes obtained at the DNA biosensors; Figure S11: Washing of the DNA layer after interaction with [Cu(TPMA)(PD)]_2+_; Figures S12–S15: The relationship between the oxidation and reduction wave peak currents versus the scan rate, and the square root of the scan rate, at the DNA biosensor; Figures S16–S19: The stability of the complexes at the DNA biosensor; Tables S3–S6: DNA nuclease efficacy of copper complexes. References [17,37,45,46,47,48,49,50,52,61,64,65] have been cited in the Supplementary Materials.

## Sample Availability

Samples of the compounds are available upon request.
