# Supplementary material for: Mapping the DNA Damaging Effects of Polypyridyl Copper Complexes with DNA Electrochemical Biosensors
Source: Molecules. 2022 Jan 19;27(3):645. doi: 10.3390/molecules27030645 (PMC8838702; doi:10.3390/molecules27030645)
Supplement: Supplementary file 1 [file molecules-27-00645-s001.zip › molecules-1507238-supplementary.pdf]

Supporting Materials

Accompanying the manuscript

Mapping DNA Interactions and Quantitative Damaging Effects of Polypyridyl-based Copper Complexes via DNA Electrochemical Biosensors

Anna Banasiak [1], Nicolo Zuin Fantoni [2,3], Andrew Kellett [3,4,*], John Colleran [1,5,*]

1 Applied Electrochemistry Group, FOCAS Institute, Technological University Dublin, Camden Row, D08 CKP1 Dublin 8, Ireland; annabanasiak89@gmail.com

2 Department of Chemistry, University of Oxford, Oxford OX1 3TA, UK; nicolo.fantonizuin@chem.ox.ac.uk

3 School of Chemical Sciences and National Institute for Cellular Biotechnology, Dublin City University, Glasnevin, D09 NR58 Dublin 9, Ireland

4 Synthesis and Solid-State Pharmaceutical Centre, School of Chemical Sciences, Dublin City University, Glasnevin, D09 NR58 Dublin 9, Ireland

5 Central Quad Grangegorman, School of Chemical and Pharmaceutical Sciences, Technological University Dublin, D07 H6K8 Dublin 7, Ireland

* Correspondence: andrew.kellett@dcu.ie (A.K.); john.colleran@tudublin.ie (J.C.); Tel: +353-1-700-5461 (A.K.); +353-1-220-5562 (J.C.)

List of Contents:

S-1: DNA biosensor preparation
S-2: The effect of acetonitrile on the DNA layer of the DNA biosensor
S-3: Electrochemical response of $[Cu(TPMA)(phen)]^{2+}$, $[Cu(TPMA)]^{2+}$ and $[Cu(TPMA)(PD)]^{2+}$ at the gold electrode
S-4: Reduction of the pyridine ring
S-5: Electrochemical parameters for the Copper Complexes obtained at the DNA biosensors
S-6: Washing of the DNA layer after interaction with $[Cu(TPMA)(PD)]^{2+}$
S-7: The relationship between the oxidation and reduction wave peak currents versus the scan rate, and the square root of the scan rate, at the DNA biosensor
S-8: The stability of complexes at the DNA biosensor
S-9: DNA Nuclease Efficacy of Copper Complexes

S-1: DNA biosensor preparation

Freshly cleaned disc gold electrodes were immersed in 0.5 M phosphate buffer, pH 7.0, containing 0.4 μM double-stranded thiolated Oligo DNA and left overnight. DNA strands modified with the thiol group promote the creation of self-assembled monolayer on the gold electrode surface.[61,65] The DNA strands were encouraged to adopt a more perpendicular position by applying a small cathodic potential (−0.6 V vs. SCE) at the DNA-modified electrodes for 30 seconds. The DNA-modified electrodes were then immersed in a 1 mM p-toluenethiol solution for one hour to backfill 'pin holes' present between Oligo DNA strands after immobilisation. Backfilling spaces between DNA strands prevents any non-specific interactions between the compounds and the gold electrode surface.

The glassware used during the immobilisation step was coated with chlorotrimethylsilane to avoid adsorption of Oligo DNA strands onto the glass surface—such adsorption could affect the concentration of DNA in solution and, consequently, the optimised immobilisation procedure.[64]

S-2: The effect of acetonitrile on the DNA layer of the DNA biosensor.

The investigated complexes are not immediately soluble in aqueous solutions. Stock solutions of each complex were prepared in acetonitrile (ACN) and then added to phosphate buffer (electrolyte during the electrochemical analysis). ACN as an organic solvent can denature DNA. To examine if the addition of small ACN aliquots affects the DNA layer immobilised on the electrode surface, control measurements were performed.

The DNA biosensor was immersed in 10 mL 0.1 M phosphate buffer (PB), pH 7.0, and CV was carried out over the potential range +0.5 V to −0.6 V vs. SCE. The amount of ACN added to PB with the compounds is 20 μL; hence, the effect of that volume and larger volumes (50 μL and 100 μL) were examined.

No change in the current at the DNA biosensor was observed after the addition of acetonitrile. Hence, in the presence of ACN (up to 100 μL) the DNA layer was stable (Figure S1).

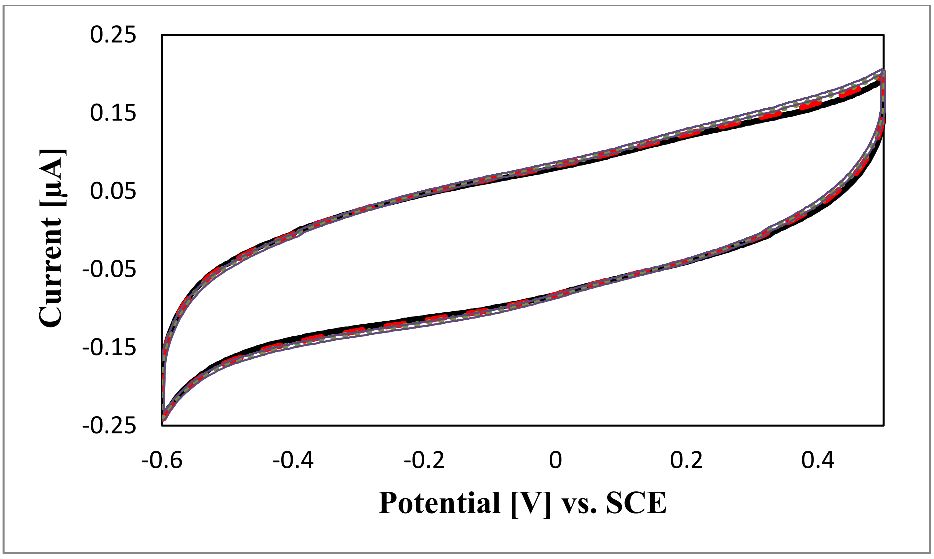

Figure S1. Cyclic voltammograms registered at the DNA biosensor in 0.1 M phosphate buffer, pH 7.0, (black solid trace), and after addition of 20 μL (red dashed trace); 50 μL (green dotted trace) and 100 μL of ACN (violet double trace); scan rate: 100 mV s^{-1}.

S-3: Electrochemical behaviour of $[Cu(TPMA)(phen)]^{2+}$, $[Cu(TPMA)]^{2}$ and $[Cu(TPMA)(PD)]^{2+}$ at bare gold electrodes

The electrochemical profile of $[Cu(TPMA)(phen)]^{2+}$ exhibits two reduction peaks (C_1, C_2) and one oxidation peak (A_1) at gold electrodes in 0.1 M PB, pH 7.0, over the potential range +0.5 to −0.6 V vs. SCE (Manuscript, Figure 1a).

To examine how the individual peaks are correlated with each other, the measurements in narrower potential windows were performed. When the CV measurement was carried out from +0.5 V to −0.4 V vs. SCE, the C_1 and A_1 peaks were barely visible at −0.22 V and +0.33 V vs. SCE, respectively (Figure S2). The redox peaks, C_1/A_1, appeared close to events observed in electrolyte (PB) only so further investigation was required.

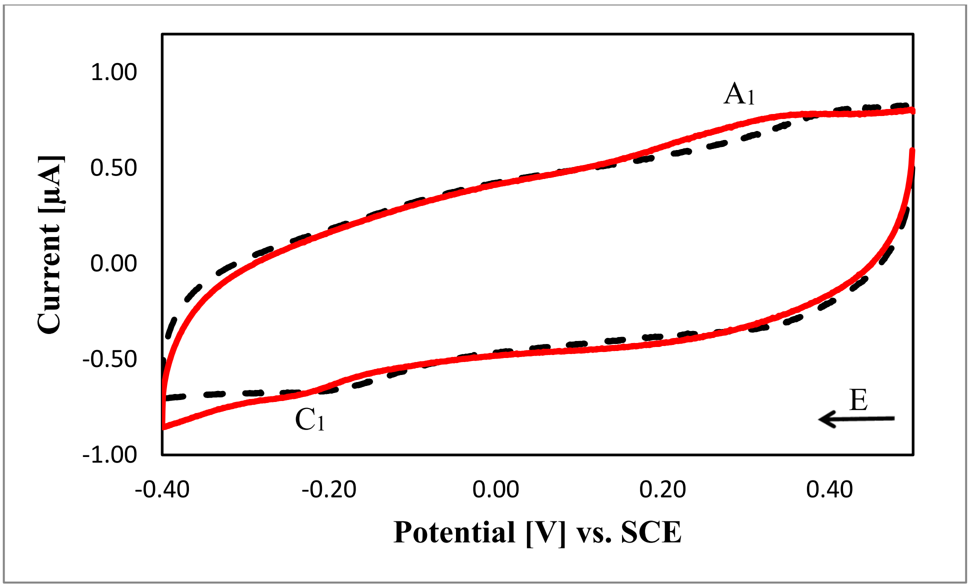

Figure S2. Cyclic voltammograms registered from +0.5 V to −0.4 V at the bare gold electrode in 0.1 M PB, pH 7.0, (black dashed trace) and in 20 µM of $[Cu(TPMA)(phen)]^{2+}$ (red solid trace). Voltammograms obtained at a scan rate of 100 mV s^{-1}.

The $[Cu(TPMA)(phen)]^{2+}$ was also cycled in more negative potentials, that is, from −0.3 V to −0.6 V vs. SCE (Figure S3). In this window, the C_2 peak did not appear, suggesting that the C_2 redox process occurs only when it is preceded by a C_1 reduction. Hence, C_1 and then also A_1 are associated with the redox reaction of the complex and are not due to the buffer (otherwise C_2, as an independent process, should be visible in a shorter potential window).

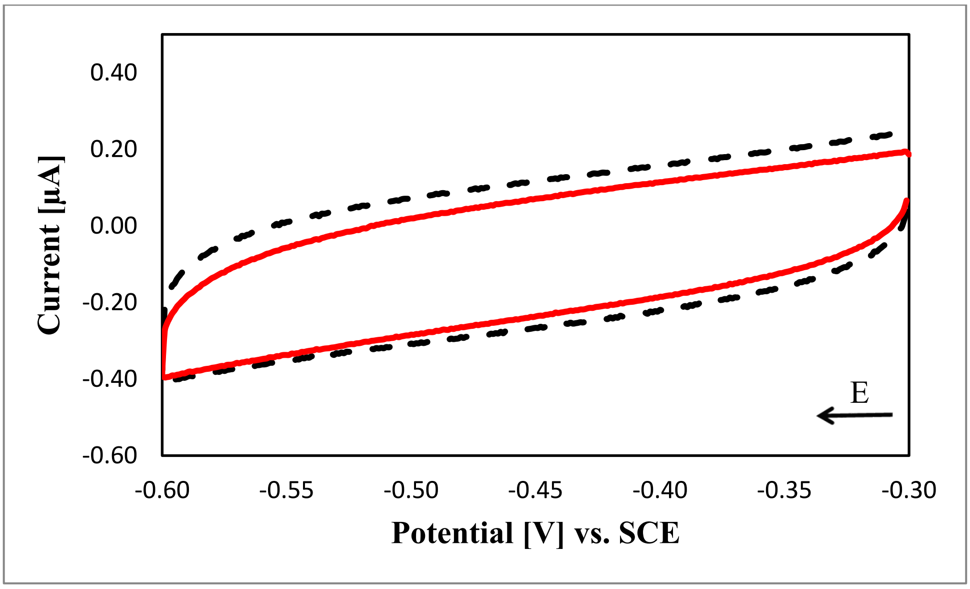

Figure S3. Cyclic voltammograms registered from −0.3 V to −0.6 V vs. SCE at the bare gold electrode in 0.1 M PB, pH 7.0, (black dashed trace) and in 20 µM of $[Cu(TPMA)(phen)]^{2+}$ (red solid trace). Voltammograms obtained at a scan rate of 100 mV s^{-1}.

Hence, the C_1/A_1 redox process is coupled and can be associated with the reduction and oxidation of copper-based complex as follows:

$$[Cu(TPMA)(phen)]^{2+} + e^- \rightarrow [Cu(TPMA)(phen)]^{+} \quad (C_1)$$
$$[Cu(TPMA)(phen)]^{+} \rightarrow [Cu(TPMA)(phen)]^{2+} + e^- \quad (A_1)$$

The C_2 peak can be then associated with the further reduction of the complex as this peak is dependent of the C_1 process.

To determine if C_2 is associated with the presence of the TPMA ligand in the structure of the complex, the electrochemical response of $[Cu(TPMA)]^{2+}$ at the bare gold electrode was examined (Figure S4).

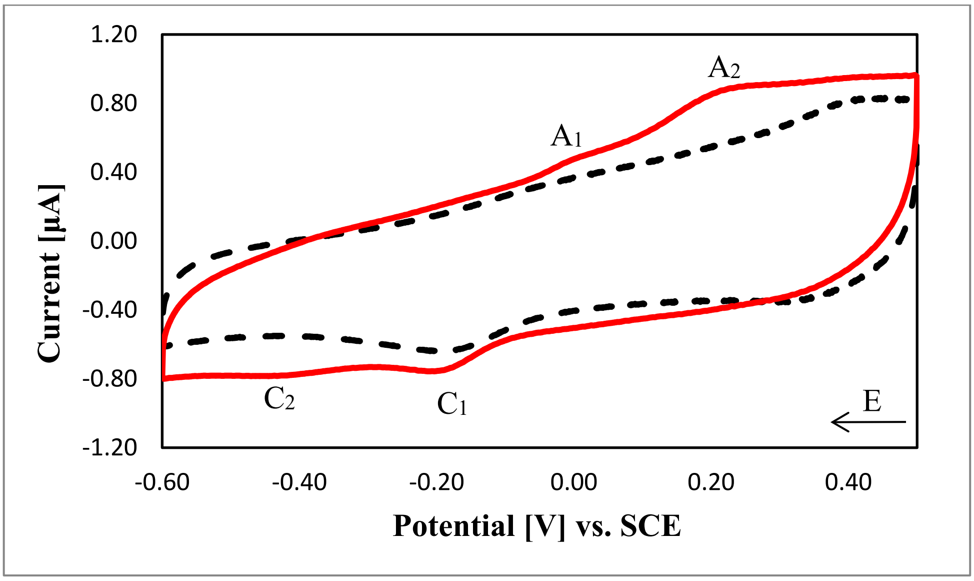

Figure S4. Cyclic voltammograms registered at the bare gold electrode in 0.1 M PB, pH 7.0, (black dashed trace), and 20 µM of $[Cu(TPMA)]^{2+}$ (red solid trace). Voltammograms obtained at a scan rate of 100 mV s^{-1}.

Two sets of redox waves were observed for $[Cu(TPMA)]^{2+}$, with the more negative redox couple, C_2/A_2, at −0.46 V (C_2) and +0.01 V (A_2). The C_2 peak was recorded at a similar potential to the C_2 peaks observed in the other copper TPMA complexes. This redox wave is then likely associated with the presence of TPMA ligand in the complex structure. Thus, C_2/A_2 can be attributed to the $[Cu(TPMA)]^{2+} \leftrightarrows [Cu(TPMA)]^{+}$ redox reaction – with associated potentials comparable to those reported in polyelectrolyte.[45] Since the TPMA ligand alone (not bound in the complex) is not electroactive under these conditions (data not shown but the TPMA ligand does not possess any electroactive groups in the structure), the second redox couple can not be attributed to this ligand. It was reported that in aqueous electrolyte, copper complexes of TPMA exist in a five-coordinated geometry.[17] In aqueous solution the fifth coordination site is likely to be a copper-oxo species, possibly occupied by a H_2O molecule, and the C_1/A_1 redox couple at −0.21 V (C_1) and +0.22 V (A_1) likely describes this electrochemical process.

$[Cu(TPMA)(PD)]^{2+}$ exhibits a more complicated electrochemical profile than the other complexes. Four reduction (C_1, C_2, C_3, C_4) peaks and three oxidation (A_1, A_2, A_3) peaks were observed for this complex at the gold electrode in 0.1 M PB, pH 7.0, in the potential range +0.5 to −0.6 V vs. SCE (Manuscript, Figure 1b, included here for clarity, Figure S5a).

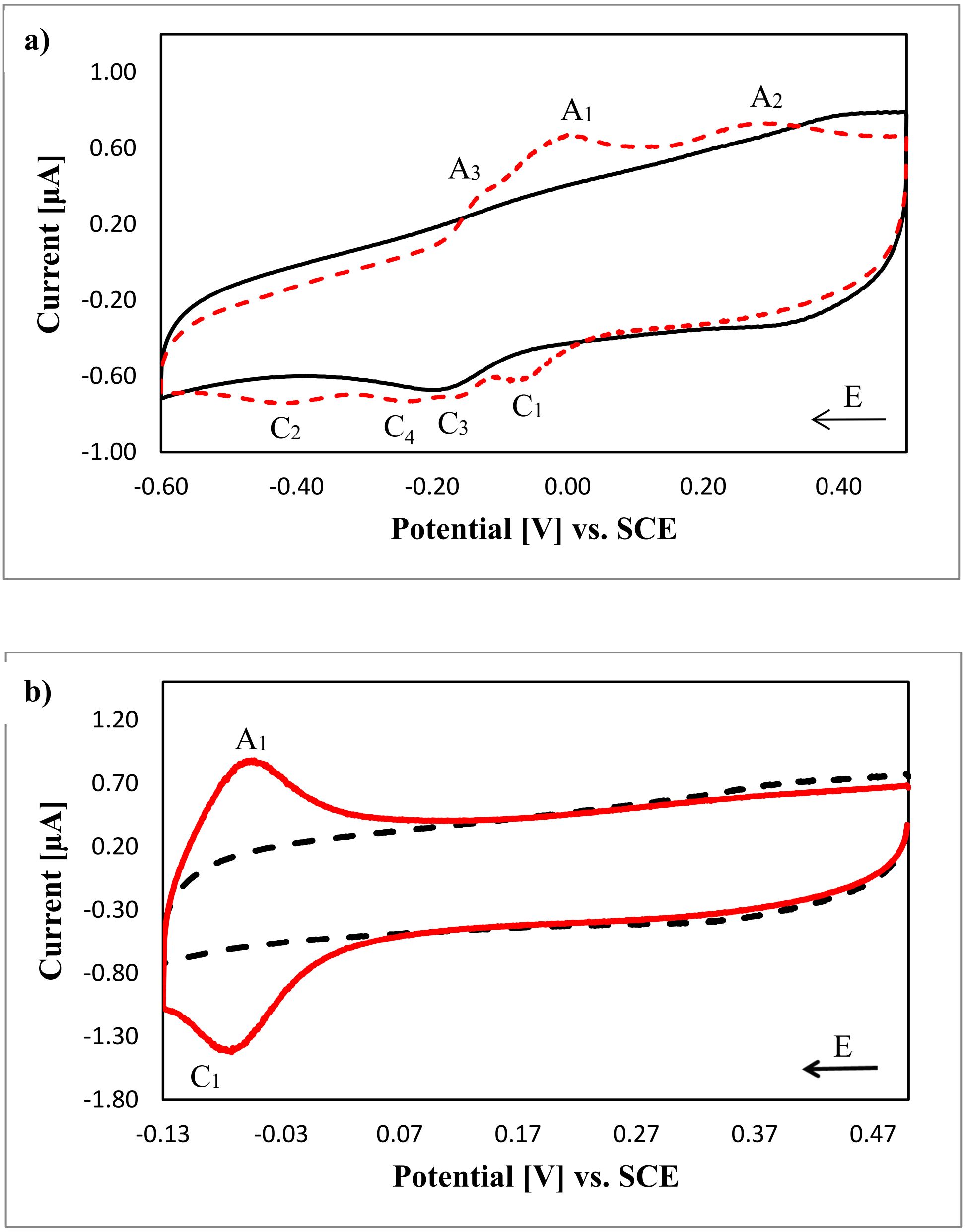

Figure S5. a) Typical cyclic voltammograms registered from +0.5 V to −0.6 V vs. SCE at bare gold electrodes in 0.1 M PB, pH 7.0 (black trace) and in 20 µM of $[Cu(TPMA)(PD)]^{2+}$ (red dotted trace); b) representative cyclic voltammograms registered from +0.5 V to −0.13 V vs. SCE at a bare gold electrode in 0.1 M PB, pH 7.0, (black dashed trace) and in 20 µM of $[Cu(TPMA)(PD)]^{2+}$ (red solid trace). Scan rates, 100 mV s^{-1}.

A shorter potential window reveals that the A_2 peak is not coupled to the C_1 reduction process (Figure S5b). When the potential window was extended slightly to more negative values (−0.3 V), a second redox couple, pre-peaks C_3/A_3, was observed at −0.16 V (C_3) and +0.003 V (A_3) (Figure S6).

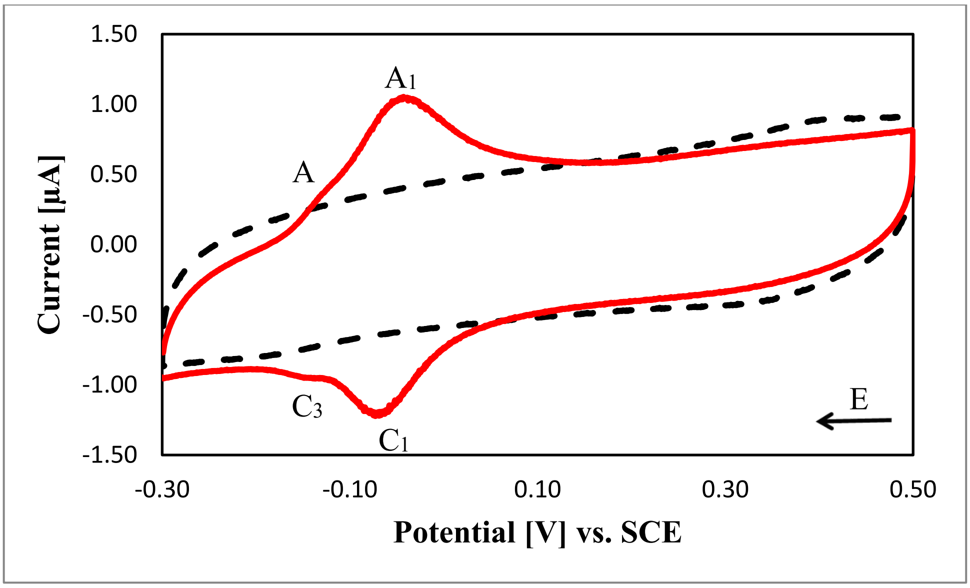

Figure S6. Cyclic voltammograms registered from +0.5 V to −0.3 V vs. SCE at a scan rate of 100 mV s^{-1} at bare gold electrodes in 0.1 M PB, pH 7.0, (black dashed trace) and in 20 μM of $[Cu(TPMA)(PD)]^{2+}$ (red solid trace).

The redox couples at these potentials can be associated with the presence of the 1,10-phen-5,6-dione (quinone) ligand in the structure. The C_1/A_1 couple describes the redox reaction for the complex ('Cu(PD) character') while the C_3/A_3 couple can be attributed to the $2e^-$ oxidation of the PD ligand, quinone to the phenol, hydroquinone. This ligand is electroactive and can undergo two-step reduction, from quinone to semiquinone and further to hydroquinone in aqueous solution.[46–50] (Figure S7).

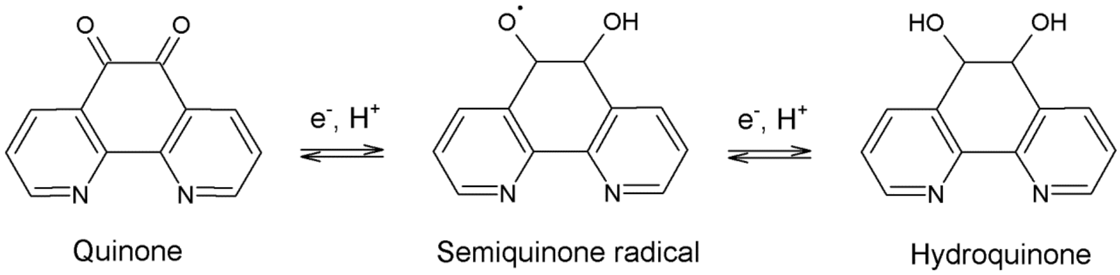

Figure S7. Scheme of quinone electrochemical reduction.[49,50]

The redox potentials of organometallic complexes of phendione depend strongly on the pH of supporting electrolyte and the type of metal present in the complex.[46–48] According to the literature,[48] the 1,10-phen-5,6-dione (quinone) ligand in a mononuclear copper complex can undergo reduction at approximately −0.1 V vs. SCE at the GCE in 0.1 M PB, pH 7.0. The redox activity observed for $[Cu(TPMA)(PD)]^{2+}$ is in good agreement with this literature value.

When the potential was cycled between −0.1 V and −0.6 V vs. SCE, the C_2 reduction peak was not observed (Figure S8), although in the wider window (Manuscript, Figure 1b) this peak is present at −0.4 V vs. SCE. This indicates that C_2 is coupled with A_2, However, when the potential was applied between +0.1 V to −0.6 V vs. SCE and the C_1/A_1, C_3/A_3 redox processes were evident, a much diminished and ill-defined C_2 reduction peak was again observed (Figure S9). The C_2 redox reaction, thus, appears to be linked to the generation of Cu^{2+}, providing additional evidence that the assigned C_2/A_2 redox couple does describe the 'Cu(TPMA) character' of the complex.

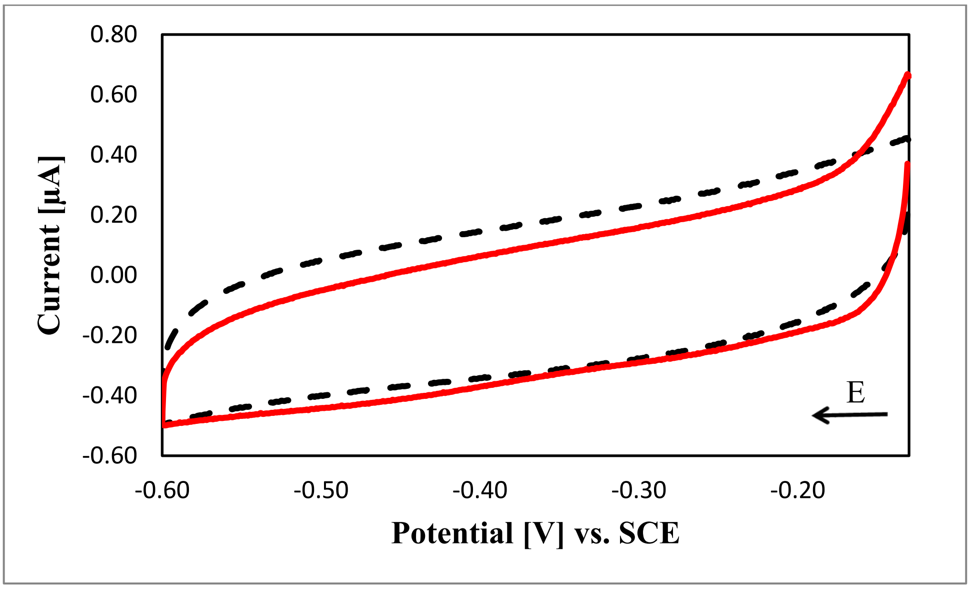

Figure S8. Cyclic voltammograms registered from −0.1 V to −0.6 V vs. SCE at a bare gold electrode in 0.1 M PB, pH 7.0, (black dashed trace) and in 20 µM of $[Cu(TPMA)(PD)]^{2+}$ (red solid trace).

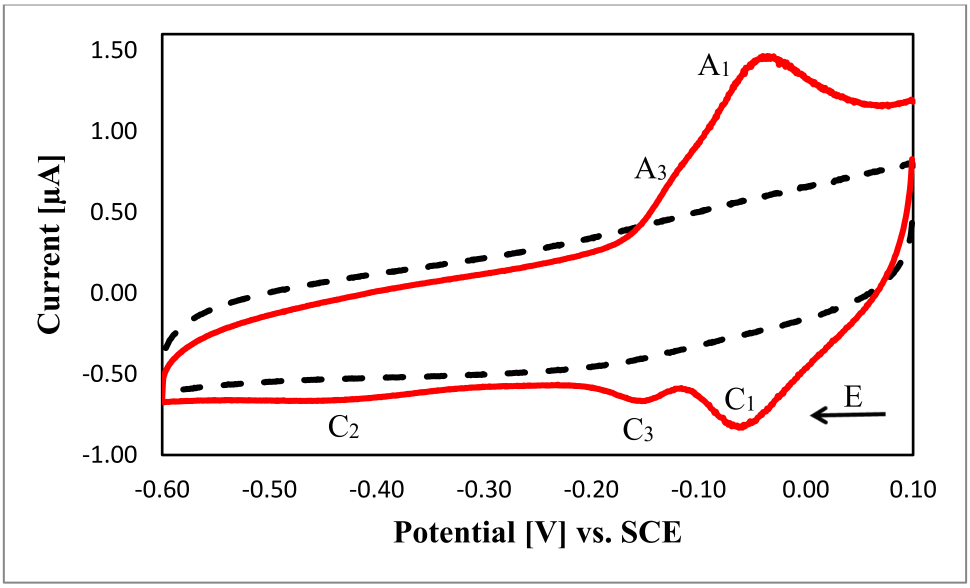

Figure S9. Cyclic voltammograms registered from +0.1 V to −0.6 V vs. SCE at a bare gold electrode in 0.1 M PB, pH 7.0, (black dashed trace) and in 20 µM of $[Cu(TPMA)(PD)]^{2+}$ (red solid trace). Voltammograms obtained at a scan rate of 100 mV s^{-1}.

Finally, after exhaustive redox cycling of the $[Cu(TPMA)(PD)]^{2+}$ complex, the reduction peak C_4 appears at −0.25 V (Figure S5a). This may be indicative of additional side reactions initiated through in-situ generation of the hydroquinone.

S-4: Reduction of the pyridine ring

The reduction of the pyridine ring at the gold electrode in 0.1 M NaF, pH 6.5, was reported to occur at −1.0 V vs. Ag/AgCl.[52] The proposed mechanism of reduction is presented in figure S10. The pyridine ring after protonation (pKa 5.2), can undergo a one electron reduction to the pyridinyl radical.

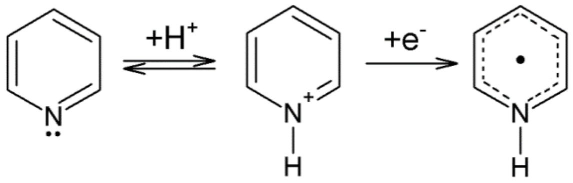

Figure S10. Scheme of pyridine ring reduction.[52]

S-5: Electrochemical parameters for the copper complexes obtained at the DNA biosensors.

Table S1: Typical redox wave potentials (C_1/A_1 and C_2/A_2) registered at DNA biosensors for the copper complexes and associated peak separation values.

**Complex name**	**$E_{p,C1}$ / $E_{p,A1}$ [V]**	**$\Delta E_{p,C1/A1}$ [mV]**	**$E_{p,C2}$ / $E_{p,A2}$ [V]**	**$\Delta E_{p,C2/A2}$ [mV]**
$[Cu(TPMA)(phen)]^{2+}$	−0.12/−0.04	80	−0.39/−0.28	110
$[Cu(TPMA)(DPQ)]^{2+}$	−0.05/+0.03	80	−0.41/−0.29	120
$[Cu(TPMA)(bipy)]^{2+}$	−0.19/+0.09	280	−0.24/−0.39	150
$[Cu(TPMA)(PD)]^{2+}$	−0.10/−0.08	20	−0.42/−0.33	90
$[Cu(TPMA)]^{2+}$	-	-	−0.42 / +0.30	120
*$[Cu(phen)_2]^{2+}$	−0.12 / −0.08	40	-	-

*Footnote: Representative data taken at 100 mV s^{-1} scan rates compared those obtained for $[Cu(phen)_2]^{2+}$. *Data obtained from reference 37.*

Table S2: Formal potentials, $E^{0'}$, for the complexes at bare and DNA modified gold electrodes, estimated from the redox waves (C_1/A_1 and C_2/A_2)

**Complex name**	**$E^{0'}_{C1/A1}$ [Bare]**	**$E^{0'}_{C1/A1}$ [DNA]**	**Shift / mV**	**$E^{0'}_{C2/A2}$ [Bare]**	**$E^{0'}_{C2/A2}$ [DNA]**	**Shift / mV**
$[Cu(TPMA)(phen)]^{2+}$	+0.06	−0.08	−140	−0.08	−0.335	−255
$[Cu(TPMA)(DPQ)]^{2+}$	+0.01	−0.01	−20	−0.115	−0.350	−235
$[Cu(TPMA)(bipy)]^{2+}$	+0.080	−0.05	−130	−0.005	−0.315	−310
$[Cu(TPMA)(PD)]^{2+}$	−0.055	−0.09	−35	−0.08	−0.375	−295
§$[Cu(TPMA)]^{2+}$	-	-	-	−0.1	−0.360	−260
*$[Cu(phen)_2]^{2+}$	−0.14	−0.10	+40	-	-	-

*Footnote: Potentials obtained at 100 mV s^{-1}scan rates. Negative shifts in the formal potentials at the DNA biosensors indicate that complexes interact with DNA through electrostatic attraction. §No ancillary N,N' ligand. *Data obtained from reference 37.*

S-6: Washing of the DNA layer after interaction with $[Cu(TPMA)(PD)]^{2+}$

The redox waves of $[Cu(TPMA)(PD)]^{2+}$ did not disappear from the DNA layer after one hour or even one-day washing in 0.1 M PB with a small amount of ACN (0.5%) but did after two days of washing (Figure S11). That indicates that the $[Cu(TPMA)(PD)]^{2+}$ is incorporated in the DNA layer very strongly or that $[Cu(TPMA)(PD)]^{2+}$ is bound to DNA covalently.

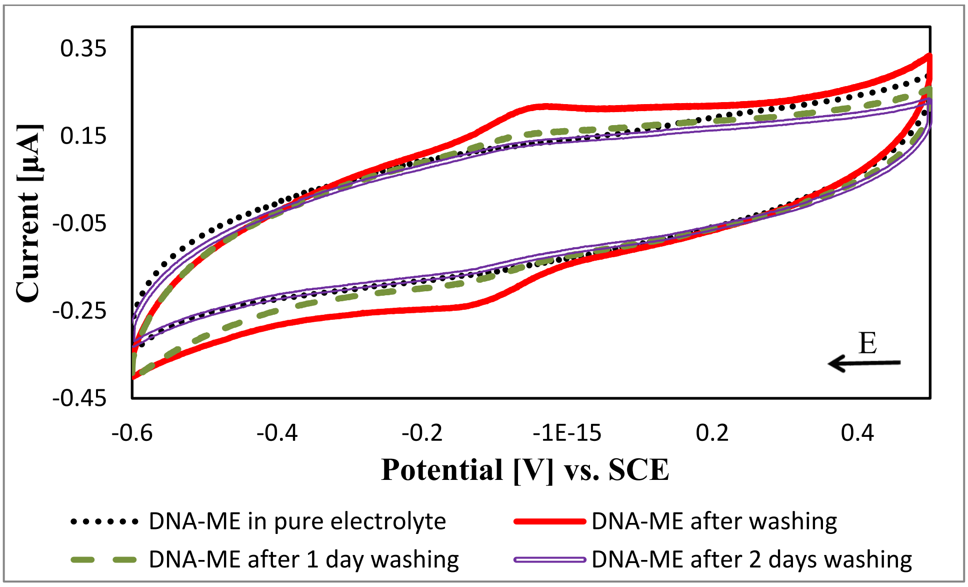

Figure S11. Typical cyclic voltammograms registered at a DNA biosensor, at a scan rate of 100 mV s^{-1}, in 0.1 M PB, pH 7.0, (black dotted trace), and after washing the DNA layer from $[Cu(TPMA)(PD)]^{2+}$ for one hour (red solid trace), one day (green dashed trace) and two days (violet double trace).

S-7: The relationship between the oxidation and reduction wave peak currents versus the scan rate, and the square root of the scan rate, at the DNA biosensor

The relationship between the oxidation and reduction peak currents versus the scan rate for $[Cu(TPMA)(phen)]^{2+}$, $Cu(TPMA)(DPQ)]^{2+}$, $[Cu(TPMA)(bipy)]^{2+}$, and $[Cu(TPMA)(PD)]^{2+}$ at the DNA biosensor were not linear, while the relationship between the peak currents and square root of scan rate was linear (Figure S12, S13, S14, S15).

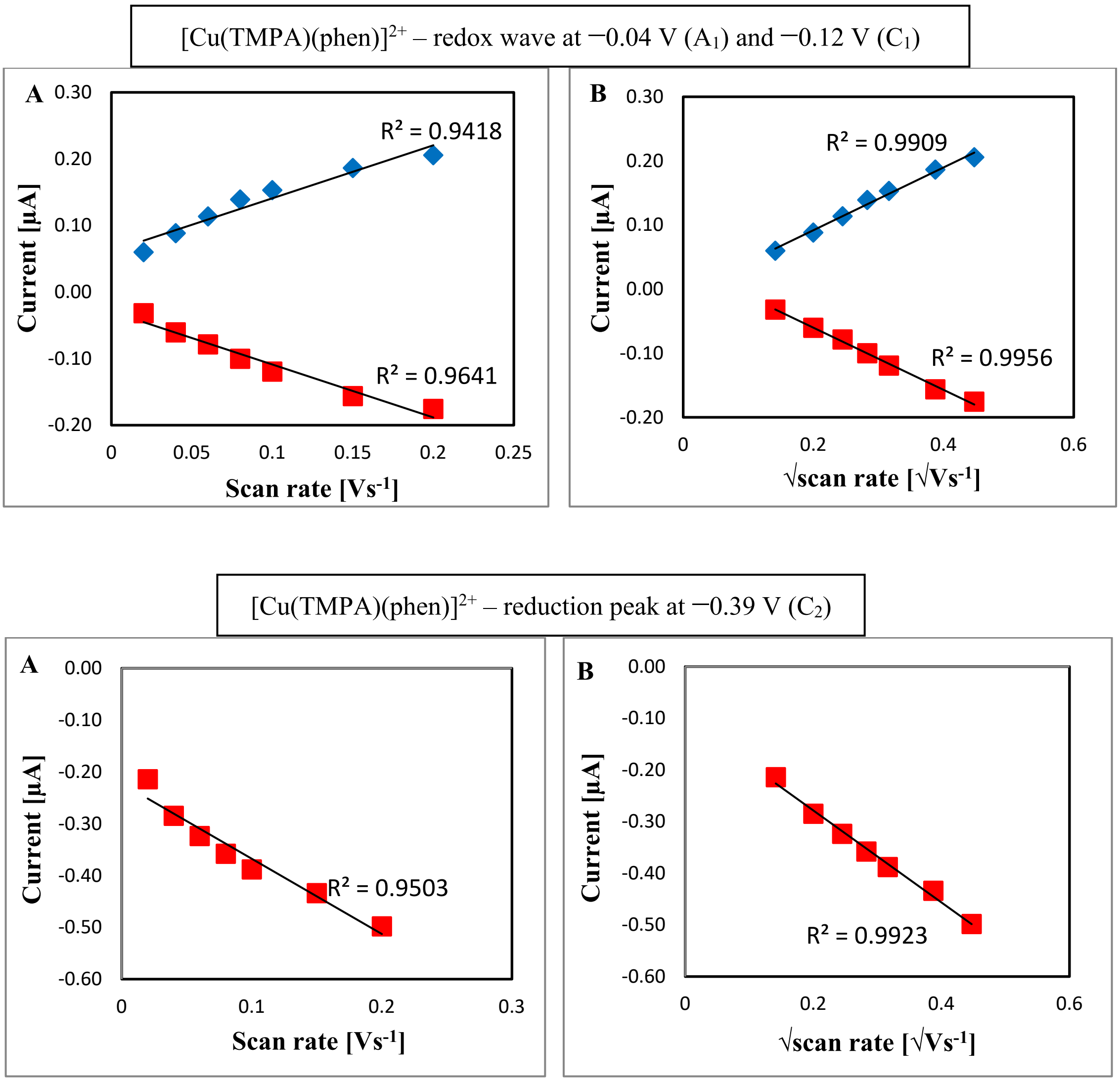

Figure S12. A plot of currents vs. A) scan rate B) square root of scan rate for the oxidation (blue squares) and reduction (red squares) of 20 µM $[Cu(TPMA)(phen)]^{2+}$ at the DNA biosensor in 0.1 M PB, pH 7.0.

[Cu(TMPA)(DPQ)]$^{2+}$ – redox wave at +0.03 V (A_1) and −0.05 V (C_1)

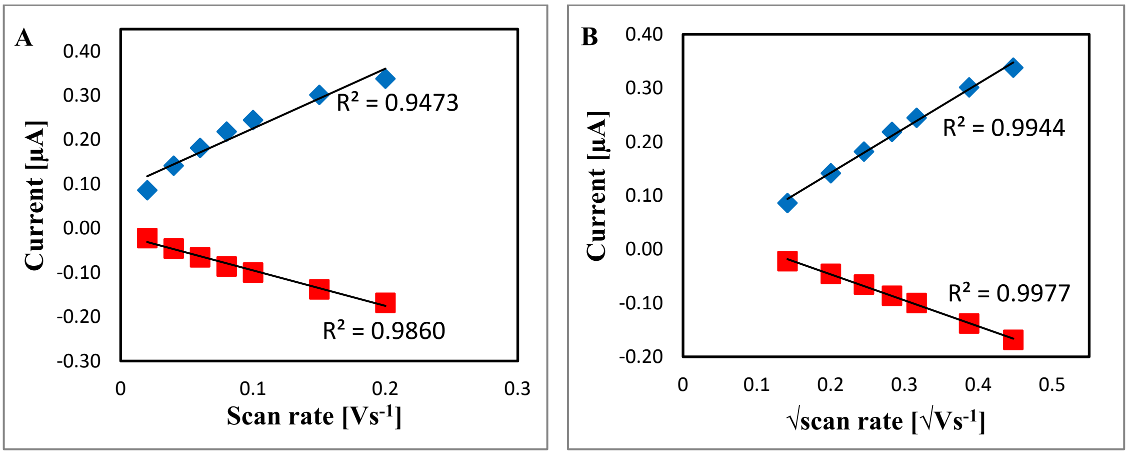

[Cu(TMPA)(DPQ)]$^{2+}$ – reduction peak at −0.41 V(C_2)

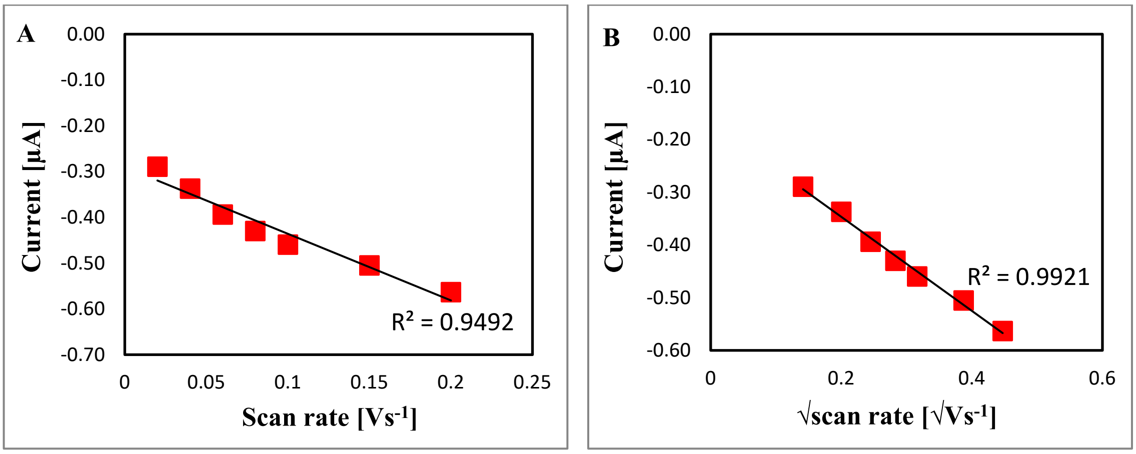

Figure S13. A plot of currents vs. A) scan rate B) square root of scan rate for the oxidation (blue squares) and reduction (red squares) of 20 µM [Cu(TPMA)(DPQ)]$^{2+}$ at the DNA biosensor in 0.1 M PB, pH 7.0.

[Cu(TMPA)(bipy)]$^{2+}$ – redox wave at −0.22 V (A_2) and −0.38 V (C_2)

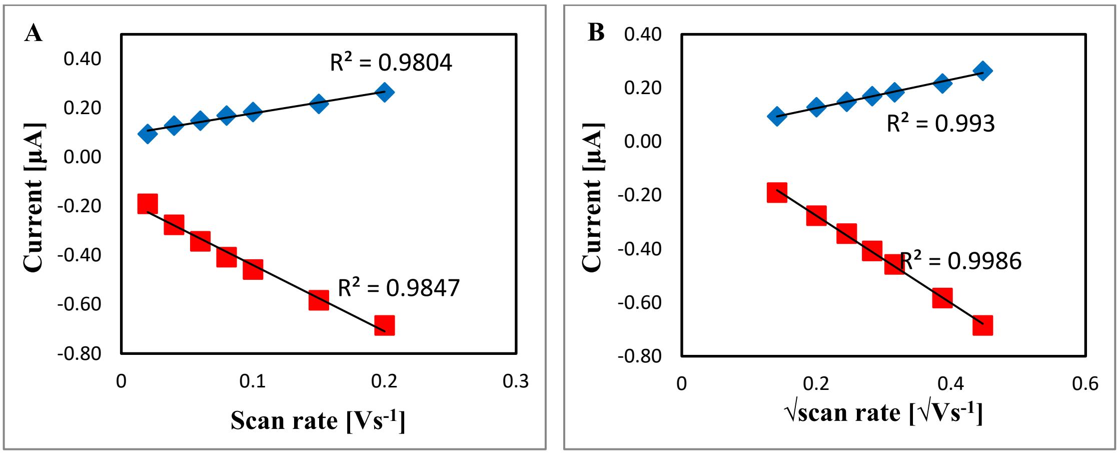

[Cu(TMPA)(bipy)]$^{2+}$ – oxidation peak at +0.11 V (A_1)

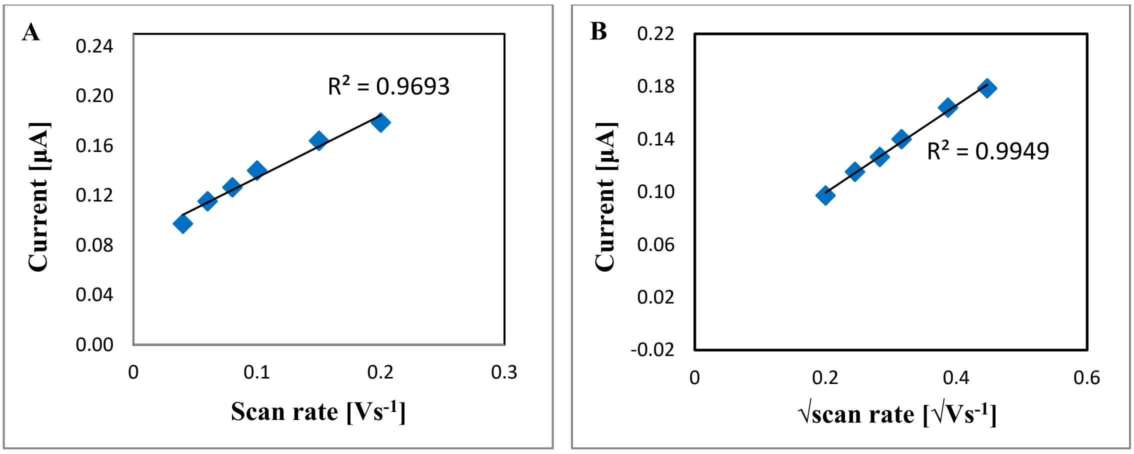

Figure S14. A plot of currents vs. A) scan rate B) square root of scan rate for the oxidation (blue squares) and reduction (red squares) of 20 µM [Cu(TPMA)(bipy)]$^{2+}$ at the DNA biosensor in 0.1 M PB, pH 7.0.

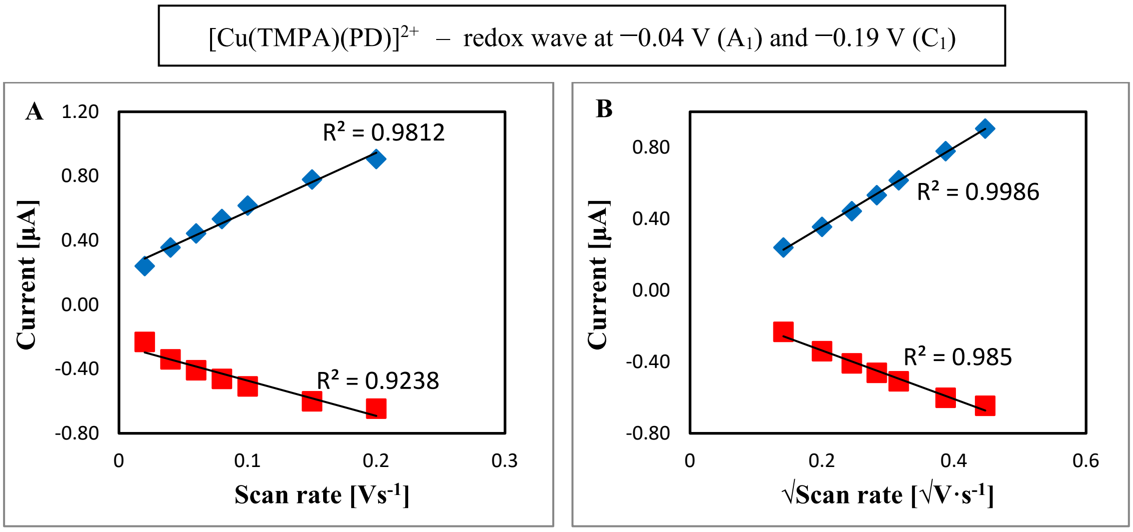

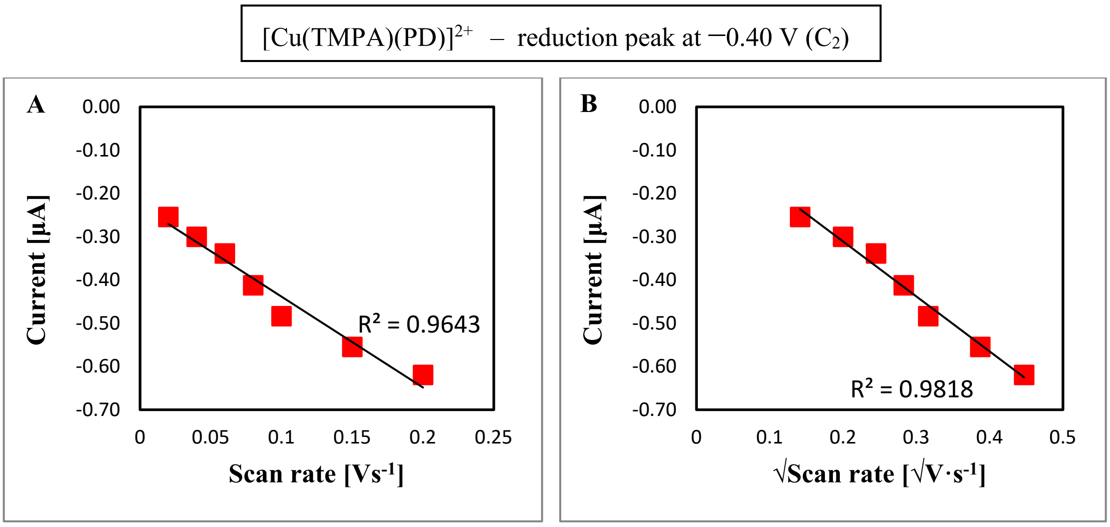

Figure S15. A plot of currents vs. A) scan rate B) square root of scan rate for the oxidation (blue squares) and reduction (red squares) of 20 µM $[Cu(TPMA)(PD)]^{2+}$ at the DNA biosensor in 0.1 M PB, pH 7.0.

Hence, the redox reactions of the copper complexes at the DNA biosensor are under diffusion control in contrast to the redox reaction of $[Cu(phen)_2]^{2+}$ which is under adsorption control.[37] This indicates that the presence of a cage ligand, TPMA, makes adsorption (intercalation within the DNA layer) less favourable than diffusion.

S-8: The stability of complexes at the DNA biosensor

The DNA biosensor was immersed in 0.1 M PB, pH 7.0, containing 20 μM of the compound (dissolved in ACN). Square wave voltammograms were registered over the potential range +0.5 V to −0.6 V vs. SCE (and reverse scan) over 2 hours for $[Cu(TPMA)(phen)]^{2+}$, $[Cu(TPMA)(DPQ)]^{2+}$ and $[Cu(TPMA)(bipy)]^{2+}$, and over 4 hours for $[Cu(TPMA)(PD)]^{2+}$. All scans were background subtracted. The SWV conditions: pulse amplitude 0.025 V; pulse width 33 ms.

The redox peaks registered for $[Cu(TPMA)(phen)]^{2+}$ (Figure S16), $[Cu(TPMA)(DPQ)]^{2+}$ (Figure S17), and $[Cu(TPMA)(bipy)]^{2+}$ (Figure S18) were stable over time.

The oxidation and reduction peak currents for $[Cu(TMPA)(PD)]^{2+}$ (Figure S19) were stable over one hour. Thereafter, the oxidation peak at −0.06 V vs. SCE increased to a maximum over three hours. The reduction peak registered at −0.06 V was then evident and a new peak at −0.17 V developed. The peaks observed at −0.17 V ($I_{p,c2}$) and at −0.36 V ($I_{p,c3}$) increased slightly over time.

The changes in the electrochemical behaviour of the $[Cu(TMPA)(PD)]^{2+}$ could be due to the disruption of the DNA strands. The initial interaction of the complex with DNA could cause slight unwinding of DNA strands that change the distance between base pairs in the DNA strands and in the size of the DNA grooves. Further insertion of the complex into the DNA strands, possibly strengthen through the creation of hydrogen bonds between the quinone groups (or corresponding oxidised phenol groups) of the complex and DNA bases, may justify the increasing reduction and oxidation peak currents with respect to irreversible disruption / modification of the immobilised DNA layer.

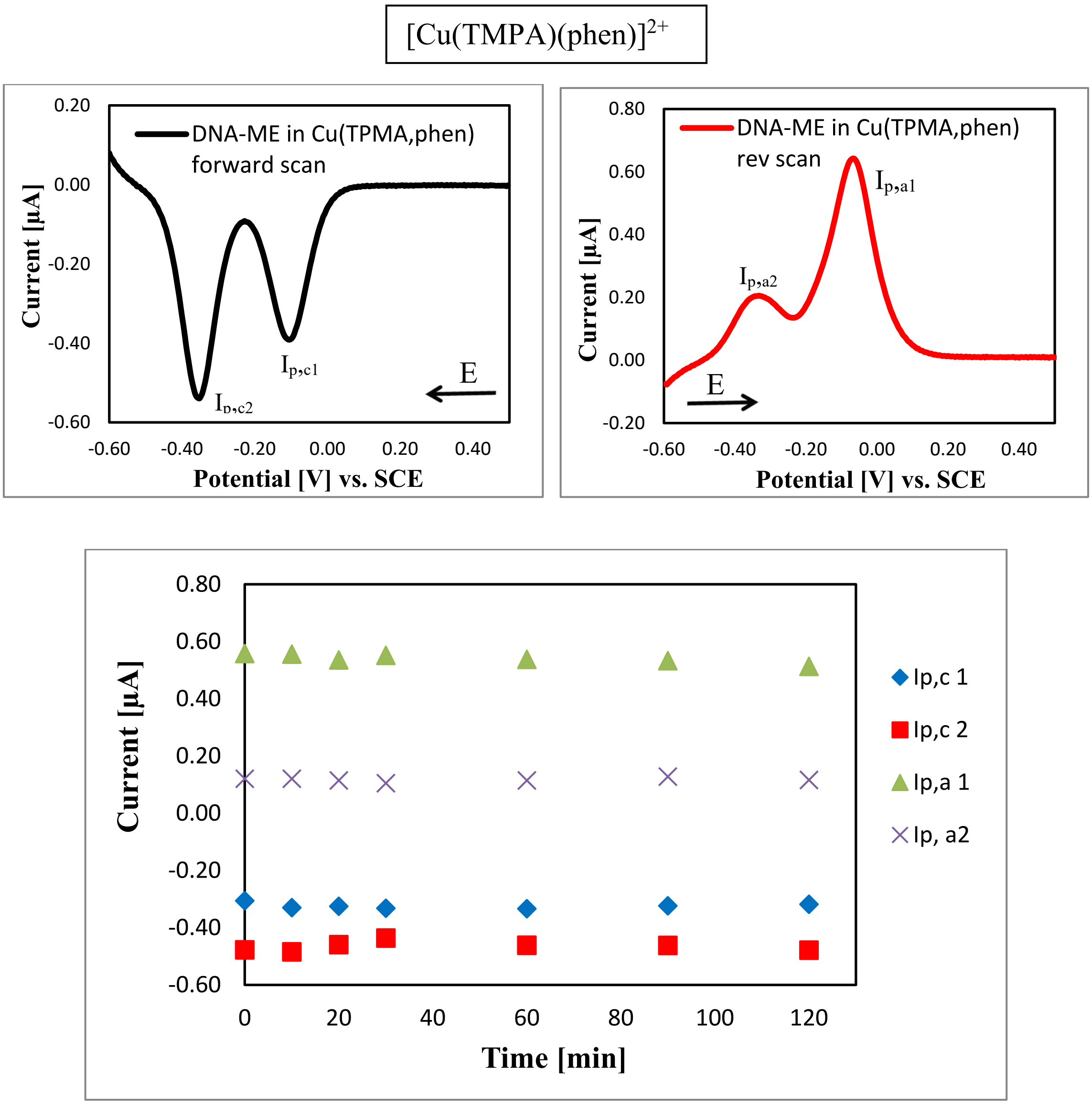

Figure S16. Typical SW voltammograms obtained in 0.1 M PB, pH 7.0, at the DNA biosensor in 20 µM $[Cu(TPMA)(phen)]^{2+}$ forward (black trace) and reverse scan (red trace) and a plot of the currents vs time for the oxidation and reduction peak currents.

[Cu(TPMA)(DPQ)]$^{2+}$

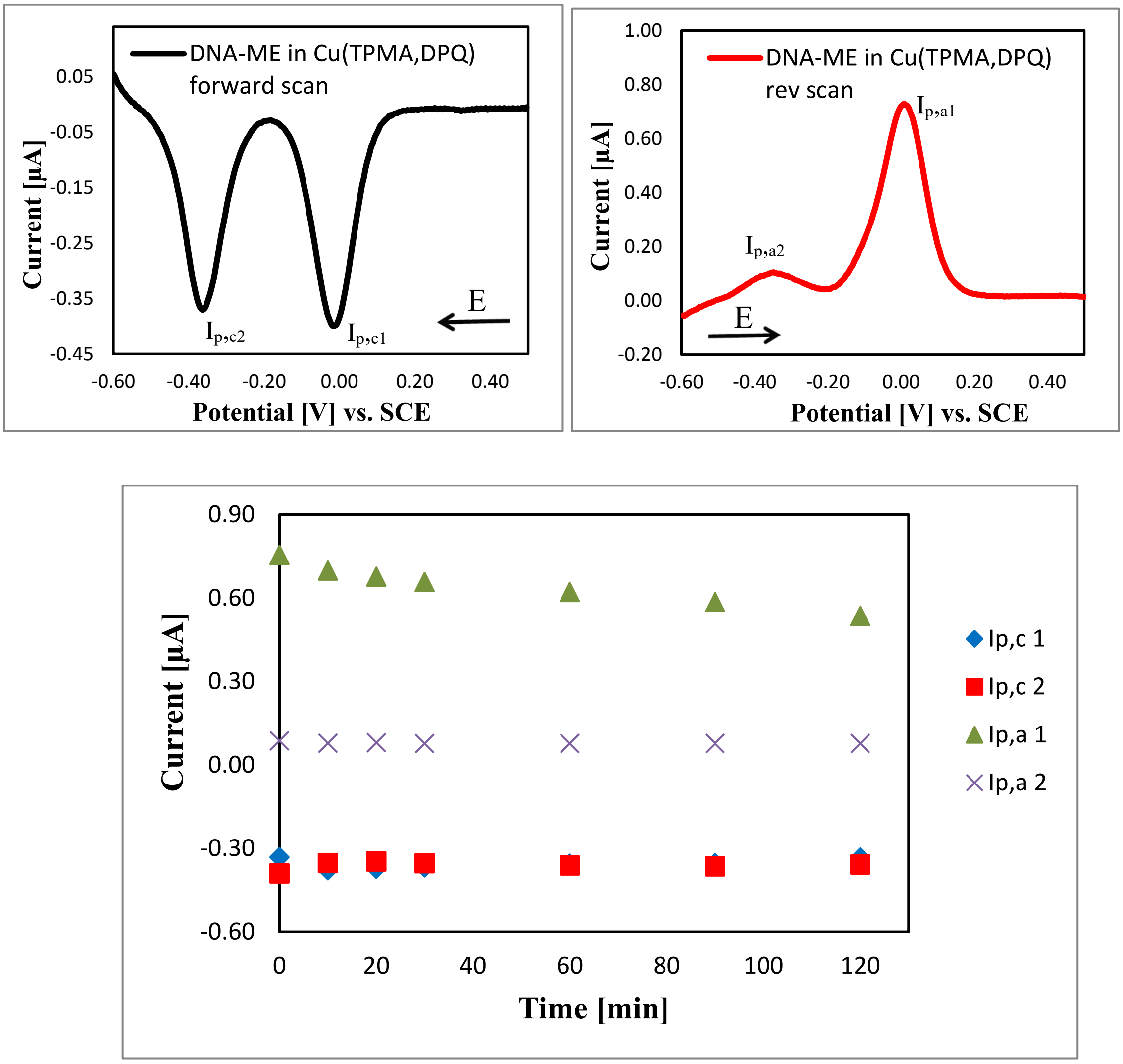

Figure S17. Typical SW voltammograms obtained in 0.1 M PB, pH 7.0, at the DNA biosensor in 20 µM [Cu(TPMA)(DPQ)]$^{2+}$ forward (black trace) and reverse scan (red trace) and a plot of the currents vs time for the oxidation and reduction peak currents.

$[Cu(TMPA)(bipy)]^{2+}$

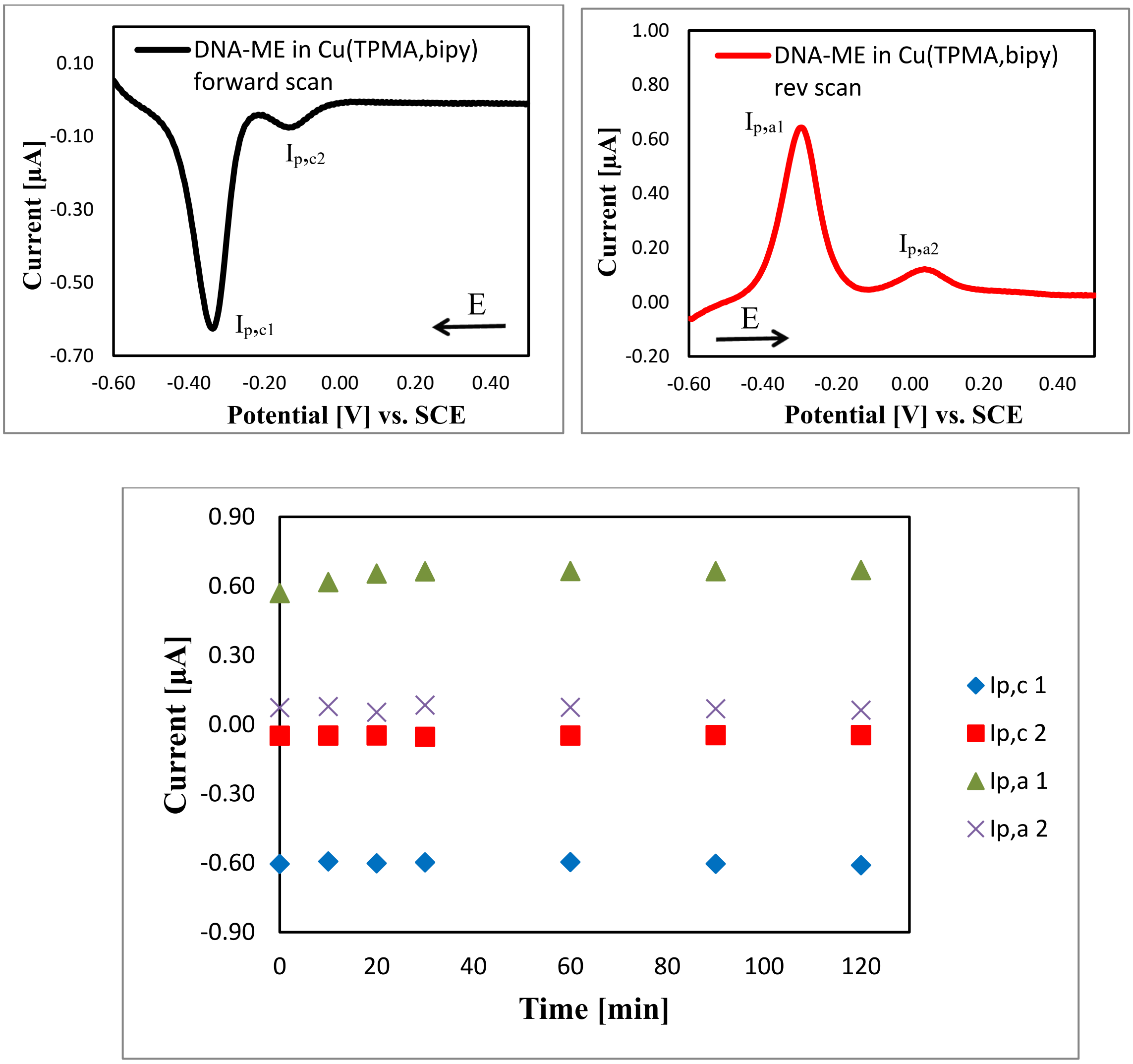

Figure S18. Typical SW voltammograms obtained in 0.1 M PB, pH 7.0, at the DNA biosensor in 20 µM $[Cu(TPMA)(bipy)]^{2+}$ forward (black trace) and reverse scan (red trace) and a plot of the currents vs time for the oxidation and reduction peak currents.

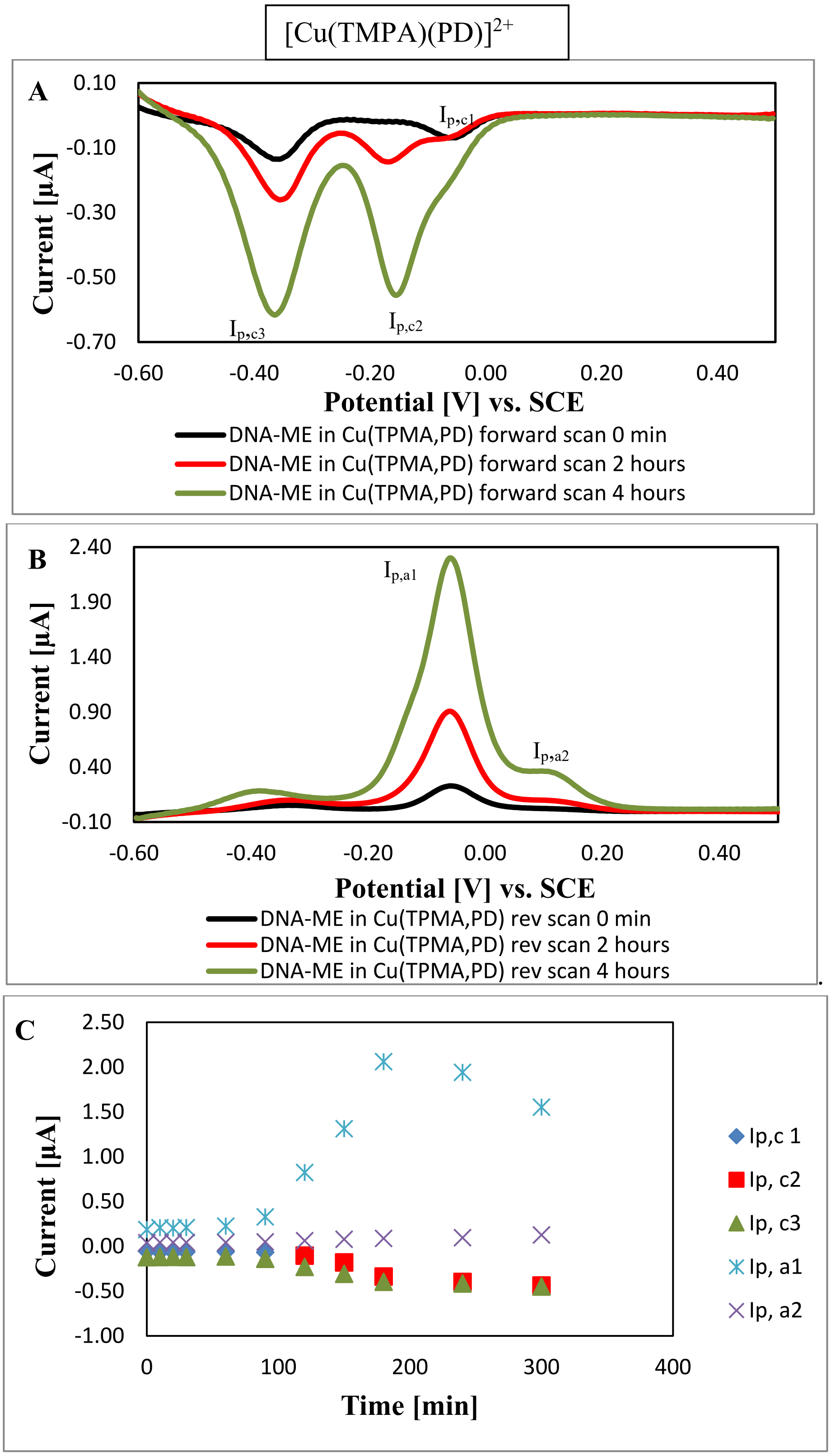

Figure S19. Typical SW voltammograms obtained in 0.1 M PB, pH 7.0, at the DNA biosensor in 20 µM $[Cu(TPMA)(PD)]^{2+}$ A) forward and B) reverse scan and C) a plot of the current vs time for the oxidation and reduction peak currents.

S-9: DNA Nuclease Efficacy of Copper Complexes

Table S3: Nuclease efficacy of $[Cu(TPMA)(phen)]^{2+}$

Experimental conditions	Average cleavage efficacy [%] (% RSD)	Average number of cleaved bases
**Control measurement**		
Supporting electrolyte with ACN only	No cleavage observed	
**Nuclease efficacy assays**		
50 µM $[Cu(TPMA)(phen)]^{2+}$ only	No cleavage observed	
10 µM $[Cu(TPMA)(phen)]^{2+}$, 1 mM AA and 1 mM H_2O_2	23.07 % (2.86 %)	6.92 bp
20 µM $[Cu(TPMA)(phen)]^{2+}$, 1 mM AA and 1 mM H_2O_2	29.51 % (5.17 %)	8.85 bp
50 µM $[Cu(TPMA)(phen)]^{2+}$, 1 mM AA and 1 mM H_2O_2	39.09 % (4.39 %)	11.73 bp

Footnote: No cleavage observed signifies that the DNA surface coverage at the DNA biosensor changed by less than 1 base pair after exposure to the nuclease assay. % RSD values are given in brackets.

Table S4: Nuclease efficacy of $[Cu(TPMA)(DPQ)]^{2+}$

Experimental conditions	Average cleavage efficacy [%] (% RSD)	Average number of cleaved bases
50 µM $[Cu(TPMA)(DPQ)]^{2+}$ only	No cleavage observed	
10 µM $[Cu(TPMA)(DPQ)]^{2+}$, 1 mM AA and 1 mM H_2O_2	20.26 % (2.49 %)	6.08 bp
20 µM $[Cu(TPMA)(DPQ)]^{2+}$, 1 mM AA and 1 mM H_2O_2	32.55 % (5.56 %)	9.76 bp
50 µM $[Cu(TPMA)(DPQ)]^{2+}$, 1 mM AA and 1 mM H_2O_2	47.75 % (0.56 %)	14.32 bp

Footnote: No cleavage observed signifies that the DNA surface coverage at the DNA biosensor changed by less than 1 base pair after exposure to the nuclease assay. % RSD values are given in brackets.

Table S5: Nuclease efficacy of $[Cu(TPMA)(bipy)]^{2+}$

**Experimental conditions**	**Average cleavage efficacy [%] (% RSD)**	**Average number of cleaved bases**
50 µM $[Cu(TPMA)(bipy)]^{2+}$ only	No cleavage observed	
10 µM $[Cu(TPMA)(bipy)]^{2+}$, 1 mM AA and 1 mM H_2O_2	5.89 % (6.32 %)	1.77 bp
20 µM $[Cu(TPMA)(bipy)]^{2+}$, 1 mM AA and 1 mM H_2O_2	6.88 % (3.69 %)	2.06 bp
50 µM $[Cu(TPMA)(bipy)]^{2+}$, 1 mM AA and 1 mM H_2O_2	25.96 % (1.55 %)	7.79 bp

Footnote: No cleavage observed signifies that the DNA surface coverage at the DNA biosensor changed by less than 1 base pair after exposure to the nuclease assay. % RSD values are given in brackets.

Table S6: Nuclease efficacy of $[Cu(TPMA)(PD)]^{2+}$

**Experimental conditions**	**Average cleavage efficacy [%] (% RSD)**	**Average number of cleaved bases**
50 µM $[Cu(TPMA)(PD)]^{2+}$ only	No cleavage observed	
10 µM $[Cu(TPMA)(PD)]^{2+}$, 1 mM AA and 1 mM H_2O_2	12.08 % (5.94 %)	3.62 bp
20 µM $[Cu(TPMA)(PD)]^{2+}$, 1 mM AA and 1 mM H_2O_2	16.22 % (0.86 %)	4.87 bp
50 µM $[Cu(TPMA)(PD)]^{2+}$, 1 mM AA and 1 mM H_2O_2	25.57 % (2.19 %)	7.67 bp

Footnote: No cleavage observed signifies that the DNA surface coverage at the DNA biosensor changed by less than 1 base pair after exposure to the nuclease assay. % RSD values are given in brackets.
